# Characterizing the selectivity of ER α-glucosidase inhibitors

**DOI:** 10.1093/glycob/cwz029

**Published:** 2019-04-29

**Authors:** Sarah O’Keefe, Quentin P Roebuck, Izumi Nakagome, Shuichi Hirono, Atsushi Kato, Robert Nash, Stephen High

**Affiliations:** 2 School of Biological Sciences, Faculty of Biology, Medicine and Health, University of Manchester, Manchester, UK; 3 School of Pharmaceutical Sciences, Kitasato University, Tokyo, Japan; 4 Department of Hospital Pharmacy, University of Toyama, 2630 Sugitani, Toyama, Japan; 5 PhytoQuest Ltd, Plas Gogerddan, Aberystwyth, Ceredigion, UK

**Keywords:** endoplasmic reticulum, glucose trimming, iminosugar inhibitors, N-linked glycosylation

## Abstract

The endoplasmic reticulum (ER) contains both α-glucosidases and α-mannosidases which process the N-linked oligosaccharides of newly synthesized glycoproteins and thereby facilitate polypeptide folding and glycoprotein quality control. By acting as structural mimetics, iminosugars can selectively inhibit these ER localized α-glycosidases, preventing N-glycan trimming and providing a molecular basis for their therapeutic applications. In this study, we investigate the effects of a panel of nine iminosugars on the actions of ER luminal α-glucosidase I and α-glucosidase II. Using ER microsomes to recapitulate authentic protein N-glycosylation and oligosaccharide processing, we identify five iminosugars that selectively inhibit N-glycan trimming. Comparison of their inhibitory activities in ER microsomes against their effects on purified ER α-glucosidase II, suggests that 3,7a-di*epi*-alexine acts as a selective inhibitor of ER α-glucosidase I. The other active iminosugars all inhibit α-glucosidase II and, having identified 1,4-dideoxy-1,4-imino-*D*-arabinitol (DAB) as the most effective of these compounds, we use *in silico* modeling to understand the molecular basis for this enhanced activity. Taken together, our work identifies the C-3 substituted pyrrolizidines casuarine and 3,7a-di*epi*-alexine as promising “second-generation” iminosugar inhibitors.

## Introduction

Widely distributed in plants, iminosugars represent a structurally diverse group of compounds, comprised of both monocyclic (piperidines and pyrrolidines) and bicyclic scaffolds (indolizines, pyrrolizidines and *nor*tropanes); and their isolation from natural sources, chemical syntheses and biological evaluation are the subject of several comprehensive reviews (Stütz 1999; Asano et al. 2000; Watson et al. 2001; Compain and Martin 2007; Asano 2008). Due to their structural mimicry of natural substrates, iminosugars have therapeutic potential in several areas of disease and have been evaluated for inhibitory activity towards a variety of α- and β-glycosidases (de Melo et al. 2006; Compain et al. 2007).

**Fig. 1.**
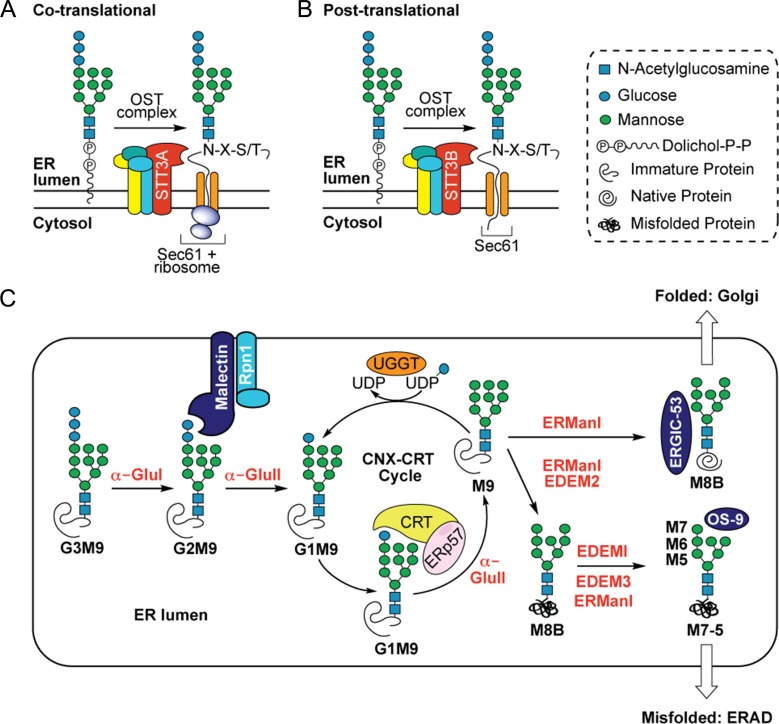
N-linked glycosylation in the ER. The multimeric oligosaccharyltransferase (OST) complex facilitates transfer of a lipid-linked glycan chain (G3M9GlcNAc_2_-) to suitable asparagine residues (N-X-S/T) of newly synthesized polypeptides via the STT3A/B catalytic subunit. (**A**) In the co-translational pathway, preassembled glycan chains are covalently attached to the growing nascent chain (via STT3A) as it emerges from the luminal side of the ER translocon (cf. Wild et al. 2018). (**B**) In the strictly post-translational pathway completed precursor proteins transit the Sec61 translocon and are N-glycosylated during or after complete translocation into the ER lumen (via STT3B). Once attached to the polypeptide chain, N-glycans (G3M9) undergo a series of trimming reactions in the ER lumen (**C**) catalyzed by α-glycosidase enzymes which sequentially remove glucose and mannose residues. In the first instance, cleavage of the terminal α-1,2-glucose residue by α-glucosidase I (α-Glu I) liberates a di-glucosylated N-glycan (G2M9) which associates with a membrane-bound lectin called malectin whilst the polypeptide associates with the OST subunit ribophorin I (Rpn1) (Qin et al. 2012). Following malectin-association, α-glucosidase II (α-Glu II) sequentially removes the two inner α-1,3-glucose residues. After the first cleavage by α-Glu II, resultant mono-glucosylated N-glycans (G1M9) are recognized by the ER luminal chaperones calreticulin (CRT) and calnexin (CNX) (not shown for simplicity), each in complex with the co-chaperone ERp57 (Oliver et al. 1999) which helps prevent aggregation and aids in polypeptide folding. The second cleavage by α-Glu II removes the innermost glucose residue generating an N-glycan comprised only of mannose residues (M9). Removal of this final glucose residue precludes further N-glycan-mediated binding to the CNX/CRT complexes but selective re-glucosylation by UDP-Glc:glycoprotein glucosyltransferase (UGGT) regenerates a G1M9 glycoform capable of rebinding CNX/CRT. Whilst exit from the CNX-CRT cycle is not fully understood, ER α-mannosidase I (ER Man I) removes a mannose residue (M8B) and, if the polypeptide has reached its native conformation, glycoproteins bearing M8B N-glycans are exported from the ER (which may be assisted by the lectin ERGIC-53) and progress through the secretory pathway. Terminally misfolded proteins, however, remain in the ER and are processed further by ER Man I and ER degradation-enhancing mannosidase-like proteins (EDEMs) (Słomińska-Wojewódzka and Sandvig 2015); i) ER Man I and/or EDEM2 recognize terminally misfolded proteins and trim a mannose residue to yield a M8B glycoform, ii) further mannose trimming of M8B by EDEMI together with EDEM3 and/or ER Man I generates M7, M6 and M5 N-glycans. Glycoproteins bearing these extensively trimmed N-glycans (assisted by the lectin OS9) are then targeted for ER-associated degradation (ERAD) (Vembar and Brodsky 2008). Monosaccharide symbols follow the SNFG (Symbol Nomenclature for Glycans) system (Varki et al. 2015).

Amongst these enzymes, α-glucosidase I (α-Glu I, EC 3.2.1.106) (Barker and Rose 2013) and α-glucosidase II (α-Glu II, EC 3.2.1.84) (Caputo et al. 2016; Satoh et al. 2016) are resident within the lumen of the endoplasmic reticulum (ER), where they process newly synthesized glycoproteins. This processing involves the sequential removal, or “trimming”, of glucose residues from the G3M9 N-glycan moiety initially added to newly synthesized glycoproteins either during or after protein synthesis (Figure 1). Trimming to G2M9 facilitates binding to malectin (Tannous et al. 2015), whilst further trimming to G1M9 enables binding to calnexin (CNX) and calreticulin (CRT) (Helenius and Aebi 2004; Tannous et al. 2015). CNX and CRT, together with the oxidoreductase ERp57, promote the correct folding and maturation of G1M9 containing glycoprotein substrates (Oliver et al. 1997; Tannous et al. 2015). Following dissociation from CNX/CRT, removal of the final glucose residue by ER α-Glu II and removal of a mannose residue by ER α-mannosidase I (ER Man I) enables the onward transport of properly folded glycoproteins to the Golgi complex where N-linked glycans may be further remodeled (Figure 1; see also Helenius and Aebi 2004; Tannous et al. 2015). In the case of partially folded/misfolded glycoproteins, selective reglucosylation by the folding sensor UDP-Glc:glycoprotein glucosyltransferase (UGGT, EC 2.4.1-) regenerates the mono-glucosylated N-glycan restoring their ability to rebind CNX/CRT. This constitutes a cycle of transient cleavage and re-addition of the innermost glucose residue (the CNX-CRT cycle) in which CNX/CRT, UGGT and ER α-Glu II work in concert to assist protein folding in the ER lumen (Figure 1). Glycoproteins that are unable to reach a native conformation are subject to sequential mannose trimming steps via ER Man I and the ER degradation-enhancing mannosidase-like proteins (EDEMs) which direct these terminally misfolded glycoproteins into a pathway(s) for ER associated degradation (ERAD) (Helenius and Aebi 2004; Ninagawa et al. 2014; Słomińska-Wojewódzka and Sandvig 2015; Tannous et al. 2015), thereby preventing their progress through the secretory pathway.

**Fig. 2.**
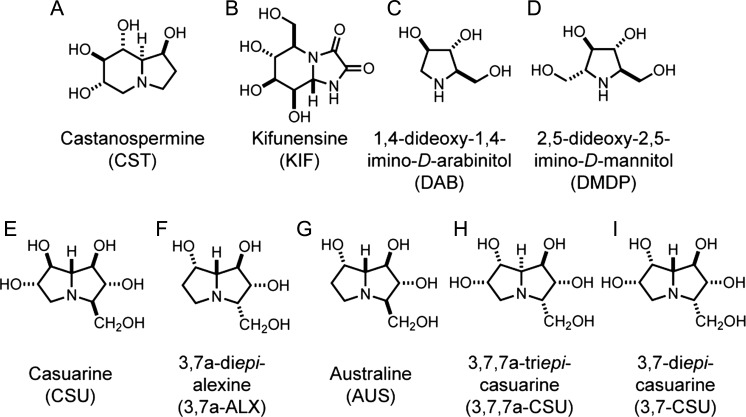
Structures of iminosugars evaluated as inhibitors of ER glycoprotein processing. The commercially available indolizidines (**A**) castanospermine (CST) and (**B**) kifunensine (KIF), and the pyrrolidines (**C**) 1,4-dideoxy-1,4-imino-*D*-arabinitol (DAB) and (**D**) 2,5-dideoxy-2,5-imino-*D*-mannitol (DMDP) provided well defined control inhibitors of ER α-glycosidases (see main text). These compounds were compared against the effects of a subset of pyrrolizidines bearing a hydroxymethyl substituent at the C-3 position; (**E**) casuarine (CSU), (**F**) 3,7a-di*epi*-alexine (3,7a-ALX), (**G**) australine (AUS) and the synthetic analogs (**H**) 3,7,7a-tri*epi*-casuarine (3,7,7a-CSU) and (**I**) 3,7-di*epi*-casuarine (3,7-CSU).

Inhibition of ER α-Glu I and ER α-Glu II precludes entry into, and/or exit from, the CNX-CRT cycle by stalling glycoproteins in an either untrimmed (G3M9) or partially trimmed (G2M9/G1M9) form. In each case, such intermediates offer scope to enhance our understanding of glycoprotein quality control processes within the ER (Araki and Nagata 2011; Tannous et al. 2015). ER α-Glu I/II inhibitors also have potential as antiviral agents which, given the paucity of broad-spectrum antivirals, warrants further investigation (Bekerman and Einav 2015). Indeed, the ER α-glucosidase inhibitors 1-deoxynojirimycin (DNJ), castanospermine (CST, Figure 2A), and their derivatives, exhibit antiviral activity towards many enveloped viruses, including: HIV (Gruters et al. 1987; Walker et al. 1987, Fleet et al. 1988); Dengue (Whitby et al. 2005; Miller et al. 2012; Watanabe et al. 2016) and Ebola virus (Chang, Warren et al. 2013). It is postulated that the abrogation of glucose trimming of viral glycoproteins, via inhibition of host ER α-Glu I/II, is sufficient to inhibit virion assembly and secretion (Chang, Block et al. 2013; Alonzi et al. 2017). However, the clinical development of DNJ, CST and their analogs has been impeded by modest reductions in viraemia and/or lack of clinical benefit (Low et al. 2014; Miller et al. 2016; Warfield et al. 2017; Ma et al. 2018). Strategies to improve the therapeutic efficacy, potency and tolerability of these compounds are ongoing (Ouzounov et al. 2002; Woodhouse et al. 2008; Watanabe et al. 2016; Ma et al. 2017, 2018; Kiappes et al. 2018).

Whilst CST, DNJ and their analogs have been studied in detail, as a bioactive class, iminosugars remain an underexplored area of chemical space (Horne et al. 2010) from which more potent inhibitors of ER α-Glu I/II may emerge. To this end, we sought to evaluate a subclass of polyhydroxylated pyrrolizidines bearing a C-3 substituent (Figures 2E–I) as ER α-Glu I/II inhibitors and we report their effects on the ER processing of N-linked glycans in a cell-free translation system supplemented with ER-derived microsomes. These compounds are compared to the well characterized α-glycosidase inhibitors CST (dual ER α-Glu I/II), (Pan et al. 1983; Kaushal et al. 1988), 4-dideoxy-1,4-imino-*D*-arabinitol (DAB, ER α-Glu II) (Asano et al. 1994) and 2,5-dideoxy-2,5-imino-*D*-mannitol (DMDP, ER α-Glu I) (Elbein et al. 1984; Asano et al. 1994) (Figures 2A, C and D) together with the mannosidase inhibitor kifunensine (KIF, ER Man I, Figure 2B) (Elbein et al. 1990). To complement these studies, we analyzed the ability of the same panel of iminosugars to inhibit the enzyme activity of purified recombinant ER α-Glu-II, determined the inhibitory constants (*K*_i_) for the four most potent inhibitors and used *in silico* modeling to establish structure-activity-relationships for these compounds.

## Results

### A subset of iminosugars alter the ER processing of N-linked glycans

**Fig. 3.**
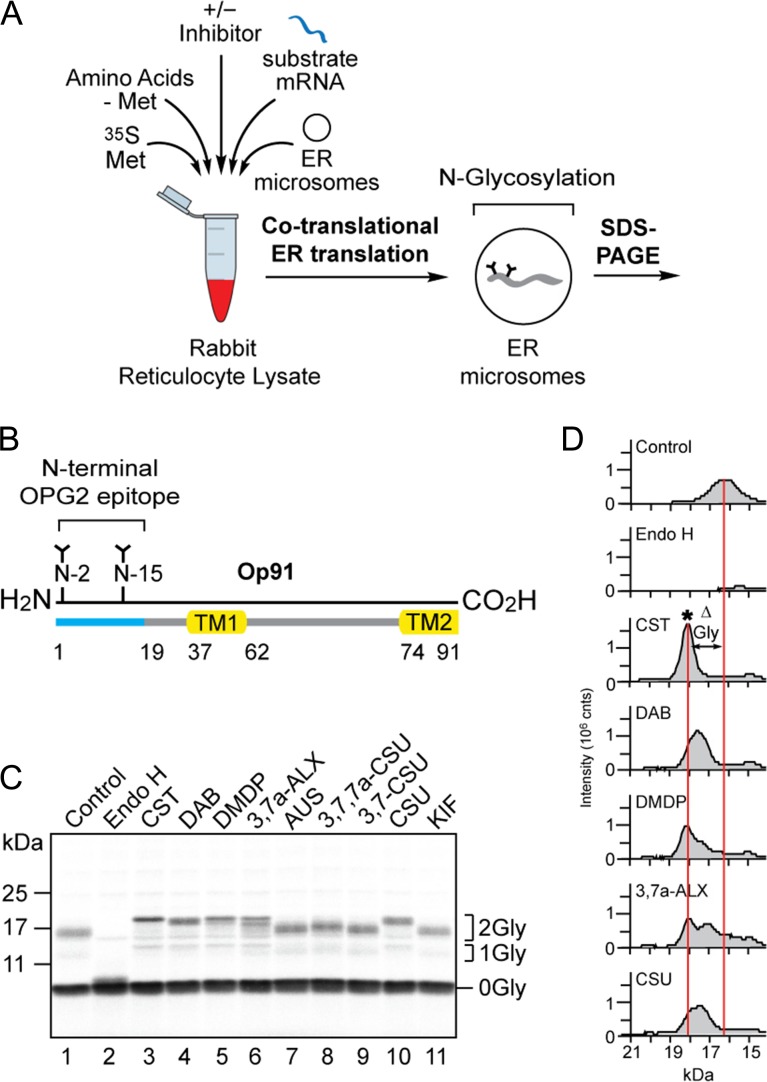
A subset of compounds alter N-glycan processing of the model glycoprotein Op91. (**A**) A schematic of the *in vitro* assay for N-glycan trimming; radiolabelled precursor proteins synthesized in the presence of ER microsomes undergo co-translational translocation, N-glycosylation and ER dependent N-glycan trimming events which can be studied by recovering the ER membrane fraction and analyzing the radiolabelled products by SDS-PAGE and phosphorimaging. (**B**) Op91 is an N-terminal fragment of bovine rhodopsin that includes the first transmembrane domain (TM1), part of the second transmembrane domain (TM2) and two endogenous sites for N-glycosylation (N2 and N15) that is efficiently inserted into ER microsomes (Crawshaw et al. 2004). (**C**) The effects of nine compounds (cf. Figure 2), each at 5 mM, on the processing of the N-glycans attached to Op91 during its synthesis (co-translationally) were assessed via a gel shift assay. Reduced migration of the major N-glycosylated species (2Gly) when compared to the non-inhibitor control (lane 1) was used to assess changes in N-glycan trimming. Treatment with Endoglycosidase H (Endo H) confirmed the identity of the N-glycosylated Op91 products (lane 2). (**D**) Gel shifts present in C were analyzed using AIDA software with peaks corresponding to the migration and signal intensity of bands. Migration profiles of the doubly N-glycosylated Op91 species generated in the presence of CST, DAB, DMDP, 3,7a-ALX and CSU were aligned with the control (C, lanes 3, 4, 5, 6 and 10 versus lane 1). Alterations in N-glycan trimming (ΔGly) as judged by changes in glycoprotein mobility are depicted between the center of the control peak and the center of the peak generated in the presence of CST which was benchmarked as the G3M9 N-glycan form and denoted by an asterisk (*).

The effects of nine compounds (Figure 2) on the ER processing of N-linked glycans were examined using a cell-free system in which radiolabelled precursor proteins are synthesized in the presence of canine pancreatic microsomes analogous to the ER (Blobel and Dobberstein 1975; Pool and Dobberstein 2011). This system faithfully recapitulates the co-translational translocation of nascent polypeptides into, and across, the ER membrane and exposes them to the N-glycosylation machinery located in the ER lumen (Walter and Blobel 1983). Suitable asparagine residues (Mononen and Karjalainen 1984; Gavel and von Heijne 1990) in the nascent polypeptide chain are covalently modified with the high mannose form of the N-glycan and these glycans then rapidly undergo initial trimming reactions characteristic of the ER (Figure 1) (Helenius and Aebi 2004). Following glycoprotein synthesis, the ER membranes were recovered by centrifugation and associated radiolabelled proteins were resolved by SDS-PAGE and visualized by phosphorimaging (Figure 3A).

In order to maximize the effect of inhibiting N-glycan trimming as assessed by changes in mobility on SDS-PAGE, we initially studied a small polypeptide with multiple N-linked glycans. To this end, the previously characterized N-terminal fragment of bovine rhodopsin (Op91) (Crawshaw et al. 2004) containing two endogenous N-glycosylation sites (hereafter denoted the OPG2 epitope) was used as a model substrate for co-translational translocation (Figure 3B). The major non-glycosylated (0Gly) and doubly N-glycosylated (2Gly) species of the Op91 polypeptide synthesized in the presence of ER-derived microsomes were identified by treatment with endoglycosidase H (Endo H) (EC 3.2.1.96), which resulted in the loss of N-glycosylated species (Figure 3C, lanes 1 and 2). Inclusion of the commercially available α-Glu I/II inhibitor CST during translation (cf. Oliver et al. 1997) resulted in a clear reduction in the mobility of the predominant 2Gly form of the Op91 polypeptide in comparison to the non-inhibitor control (Figure 3C, lanes 1 and 3). In contrast, the ER Man I inhibitor KIF (Elbein et al. 1990) had no obvious effect on the mobility of N-glycosylated species when compared to the control (Figure 3C, lanes 1 and 11). Hence, we conclude that alterations in Op91-2Gly mobility can be used to report an inhibition of glucose trimming *in vitro*.

Of the seven other compounds tested, DAB (Figure 2C), DMDP (Figure 2D) and casuarine (CSU, Figure 2E) resulted in a reduction of glycoprotein mobility that appeared comparable to CST (Figure 2A) whilst 3,7a-di*epi*-alexine (3,7a-ALX, Figure 2F) resulted in a doublet of products with reduced mobility (Figure 3C, lanes 1, 3, 4, 5, 6 and 10). The signal intensity profile of the products obtained in the presence of CST and the absence of any inhibitor provided benchmarks for the unprocessed G3M9 (Figure 3D, CST, see asterisk) and processed N-linked glycoproteins (Figure 3D, control) respectively. Profiles of the doubly N-glycosylated species of Op91 confirmed that DAB, DMDP, 3,7a-ALX and CSU also lead to a reduction in glycoprotein mobility, albeit to a varying degree (Figure 3D, see ΔGly). In contrast, any effect of 3,7,7a-tri*epi*-casuarine (3,7,7a-CSU, Figure 2H) on Op91-2Gly mobility was rather modest whilst australine (AUS, Figure 2G), and 3,7-di*epi*-casuarine (3,7-CSU, Figure 2I) had no obvious effect when compared to control and KIF treated products (Figure 3C, lanes 1, 7, 8, 9 and 11). Our finding that KIF has no obvious effect in our gel shift assay is consistent with previous reports that the inhibition of ER dependent mannose trimming is difficult to detect via changes in glycoprotein mobility on SDS-PAGE (Cannon and Helenius 1999). We therefore conclude that the alterations to the migration of Op91-2Gly that we observe in the presence of particular compounds (CST, DAB, DMDP, 3,7a-ALX and CSU) are most likely due to their inhibitory effects on glucose trimming via ER luminal α-Glu I and/or α-Glu II.

Op91-2Gly species contain two N-linked glycans making it difficult to attribute an inhibitor-dependent reduction in mobility to a precise N-glycan structure(s). However, when compared to the single major peak seen in the presence of 5 mM CST (presumed to be G3M9, see Figure 3D, CST, asterisk), a broader range of slightly faster migrating species was seen with both DAB and CSU (Figure 3D). We speculate that these products represent the accumulation of G2M9 and/or G1M9, forms of N-linked glycans consistent with the inhibition of ER α-Glu II (cf. Figure 1C). For DMDP and 3,7a-ALX the major glycoprotein species co-migrated with that of CST, although 3,7a-ALX treatment also resulted in a second prominent species of faster migration (Figure 3D). On this basis we propose that DMDP and 3,7a-ALX most likely both inhibit ER α-Glu I but do so less effectively than CST (cf. Figure 3D).

In order to establish whether the presence of multiple N-glycans influenced our ability to detect inhibitor-dependent changes in N-glycan trimming we repeated our experiments using model glycoproteins with a single N-glycosylation site (Supplementary Figure S1). In the case of the viral potassium channel Kcv, a version of the protein bearing exclusively one N-linked glycan (Supplementary Figure S1A, see 1Gly species) showed similar changes in mobility to those seen with both Op91-2Gly (Figure 3C) and OPG2Kcv (Watson et al.) a doubly N-glycosylated version of Kcv (Supplementary Figures S1Aiii-iv, see 2Gly species). Thus, CST, DAB, DMDP, 3,7a-ALX and CSU all reduced the mobility of 1Gly and 2Gly species in a similar fashion. Likewise, the trends in the changes to glycoprotein mobility were comparable when singly and doubly N-glycosylated versions of the short secretory protein preprocecropinA (Johnson et al. 2012) were analyzed by SDS-PAGE (Supplementary Figure S1B). However, subtle qualitative differences between the effects of active inhibitors were more apparent in the 2Gly form of the protein (Supplementary Figures S1Bii and S1Biv, cf. lanes 4 and 10). When migration profiles for these two additional doubly N-glycosylated glycoprotein substrates were analyzed in comparison to CST, we again found that the effects of DAB and CSU were distinct from those of DMDP and 3,7a-ALX (Supplementary Figures S2A-S2B and Figure 3D). Taken together, these data suggest that the changes in glycoprotein mobility we observe are a valid reporter for the differences in the inhibitory actions of compounds with respect to ER α-Glu I and or ER α-Glu II.

### Inhibition of N-glycan trimming is independent of when the glycan is added

Op91 provides a *bone fide* substrate for co-translational N-glycosylation (Figure 1A) since its N-terminal domain, which bears two N-glycosylation sites, is translocated into the ER lumen once the ribosome bound nascent chain arrives at the Sec61 translocon (Meacock et al. 2002). Since all of the model glycoproteins we have analyzed to date all contained N-glycosylation sites derived from the N-terminus of Op91, we next investigated whether the location, or context, of the N-linked glycans added to glycoprotein substrates influences the ability of our chosen compounds to inhibit their ER processing. To this end, the yeast secretory glycoprotein prepro-alpha-factor (ppαF), bearing three naturally occurring N-linked glycans (Supplementary Figure S1C) (Waters et al. 1988), was used as an additional model glycoprotein substrate. Band migration analysis showed that the same five compounds that altered the mobility of the previous three model substrates also resulted in the perturbation of ppαF-Gly migration (cf. Supplementary Figures S1C and S2C versus Figures 3C–D), although the presence of three N-linked glycans did increase the complexity of the products (cf. Supplementary Figure S2C).

**Fig. 4.**
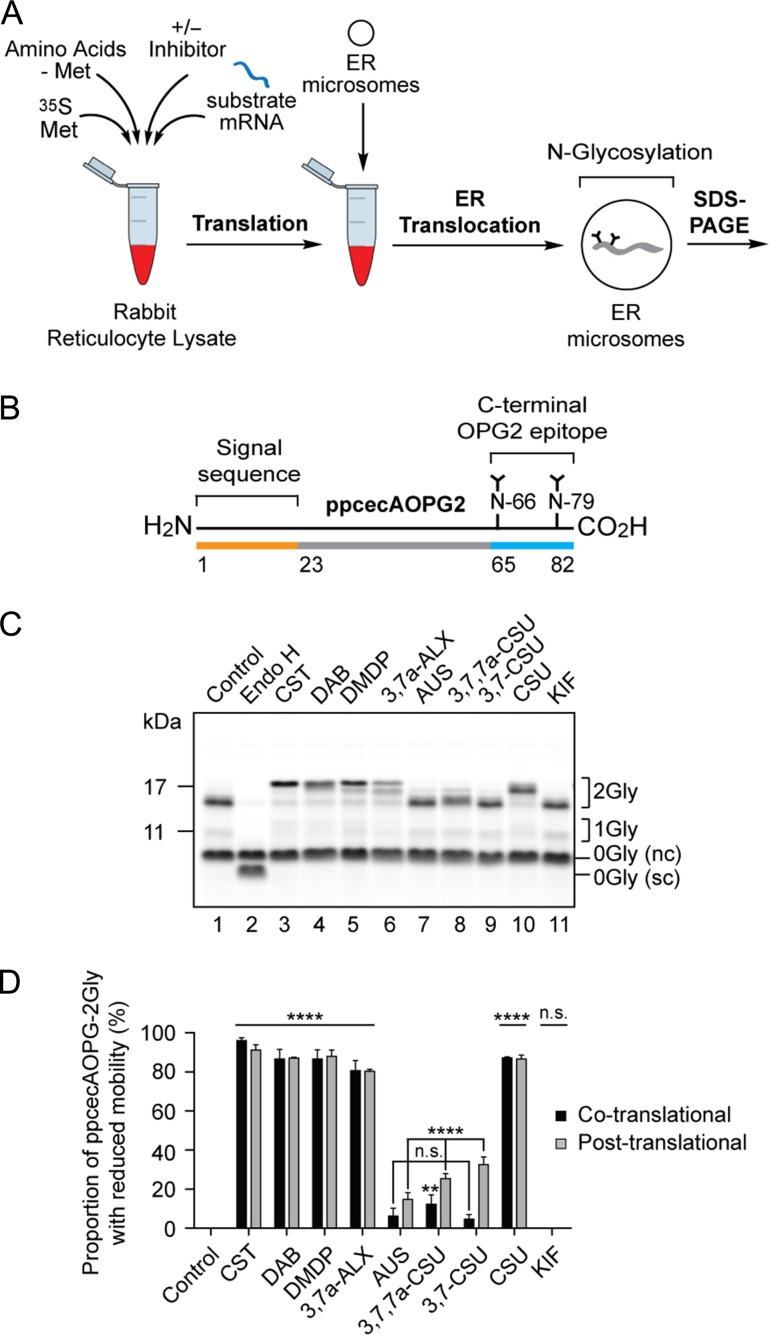
Inhibition of N-glycan trimming with a post-translationally modified glycoprotein substrate. (**A**) The radiolabelled precursor protein was synthesized as before (cf. Figure 3), but in the absence of ER microsomes. Following puromycin-induced termination of protein synthesis, completed polypeptides were incubated with ER microsomes ensuring ER translocation and subsequent N-glycosylation were strictly post-translational. (**B**) ppcecAOPG2 is a modified form of preprocecropin A containing residues 1 to 18 of bovine rhodopsin, with two sites for N-glycosylation (cf. Figure 3B), added to its C-terminus (Johnson et al. 2012). (**C**) The effect of nine compounds, each at 5 mM, on the processing of the N-glycans attached to ppcecAOPG2 post-synthesis was assessed as described in the legend to Figure 3. nc, non-cleaved signal sequence form of ppcecAOPG2; sc, signal cleaved form. (**D**) The efficiency of inhibition of N-glycan trimming in the post-translational system (light gray bars) was estimated by quantifying the signal intensity of the distinct 2Gly species with reduced mobility, as compared to the control sample, that were observed in the presence of tested compounds (e.g., Figure 4C, cf. lanes 1 and 3). The proportion of 2Gly species with reduced mobility was then expressed as percentage of the total signal for all 2Gly species. Similar calculations were performed for ppcecAOPG2-2Gly synthesized in the presence of ER microsomes where the co-translational pathway is also available (see Supplementary Figure S1Biv), and these results are included for comparison (black bars). In each case the experiments were performed in triplicate (*n* = 3). Statistical significance of compound-induced inhibition relative to the control (one-way ANOVA) was determined using Tukey’s multiple comparisons test and shown in the figure. Statistical significance comparing the levels of compound-induced inhibition between co- and post-translational mechanisms of translocation (two-way ANOVA) was determined using Sidak’s multiple comparisons test and are as follows: n.s. CST, DAB, DMDP, 3,7a-ALX, CSU; **AUS; **** 3,7,7a-CSU, 3,7-CSU. Statistical significance is given as n.s., non-significant; **, *P* < 0.01 and ****, *P* < 0.0001.

In addition to co-translational N-glycosylation, a subset of precursors and N-glycosylation sites can be modified post-translationally (Ruiz-Canada et al. 2009). We, therefore, exploited the ability of the short secretory protein preprocecropin A supplemented with a C-terminal OPG2 tag (ppcecAOPG2) to act as a well-defined post-translational substrate for N-glycosylation (Johnson et al. 2012). Following translation of ppcecAOPG2 mRNA, the separation of protein synthesis and N-glycosylation was achieved using puromycin to terminate protein synthesis prior to the addition of ER microsomes, thereby ensuring that membrane translocation proceeded via a post-translational, but Sec61-mediated, pathway (Figure 4A; see also Zimmerman et al. 1990; Johnson et al. 2012). In this way, the strictly post-translational N-glycosylation of the C-terminal tag of ppcecAOPG2 (Figure 4B) could be investigated. Strikingly, once again, the same five compounds (CST, DAB, DMDP, 3,7a-ALX and CSU) showed clear and reproducible effects on the mobility of N-glycosylated ppcecAOPG2 (Figure 4C). Quantification confirmed that all of the compounds had a comparable and statistically significant effect (Figure 4D) and we conclude that all active compounds act at a stage after N-glycan attachment, most likely by targeting α-glycosidase enzymes.

Since the model substrate ppcecAOPG2 may employ both co- and/or post-translational mechanisms of ER translocation, and their associated oligosaccharyltransferase (OST, EC 2.4.99.18) complexes for N-glycosylation (Figures 1A–B), we compared the results we obtained when the protein was synthesized in the presence (co/post) and absence (post only) of ER-derived microsomes. The quantitative effects of AUS, 3,7,7a-CSU and 3,7-CSU appeared to be more pronounced following post-translational import as compared to the co-translational system (see Figure 4D). However, we note that there is a higher proportion of untranslocated precursor (with the signal sequence intact, see product labeled 0Gly (nc)) following post-translational translocation when compared to the co-translational system (cf. Figure 4C, lane 2 and Supplementary Figure S1Biv, lane 2, 0Gly (nc) versus 0Gly (sc)). Thus, it may be that there is a reduced level of glycoprotein substrate in the ER lumen following post-translational import thereby allowing even the relatively ineffective compounds AUS, 3,7,7a-CSU and 3,7-CSU to show some degree of inhibitory effect when present at a high concentration (5 mM, see Figure 4D).

The overall conclusion from this quantitative analysis (Figure 4D) was consistent with our previous studies and indicated that CST, DAB, DMDP, 3,7a-ALX and CSU were the most active compounds as judged by changes in glycoprotein mobility. Furthermore, quantification showed that the effects of CST (Figure 2A), KIF (Figure 2B), DAB (Figure 2C), DMDP (Figure 2D), CSU (Figure 2E) and 3,7a-ALX (Figure 2F) were not statistically different between co- and post-translational pathways (Figure 4D). We therefore conclude that the inhibition of N-linked glycan processing we observe is unaffected by the pathway through which N-glycans are added to the polypeptide chain (cf. Figures 1A-1B).

### Active compounds have distinct effects on the activity of purified α-glucosidase II

**Figure 5.**
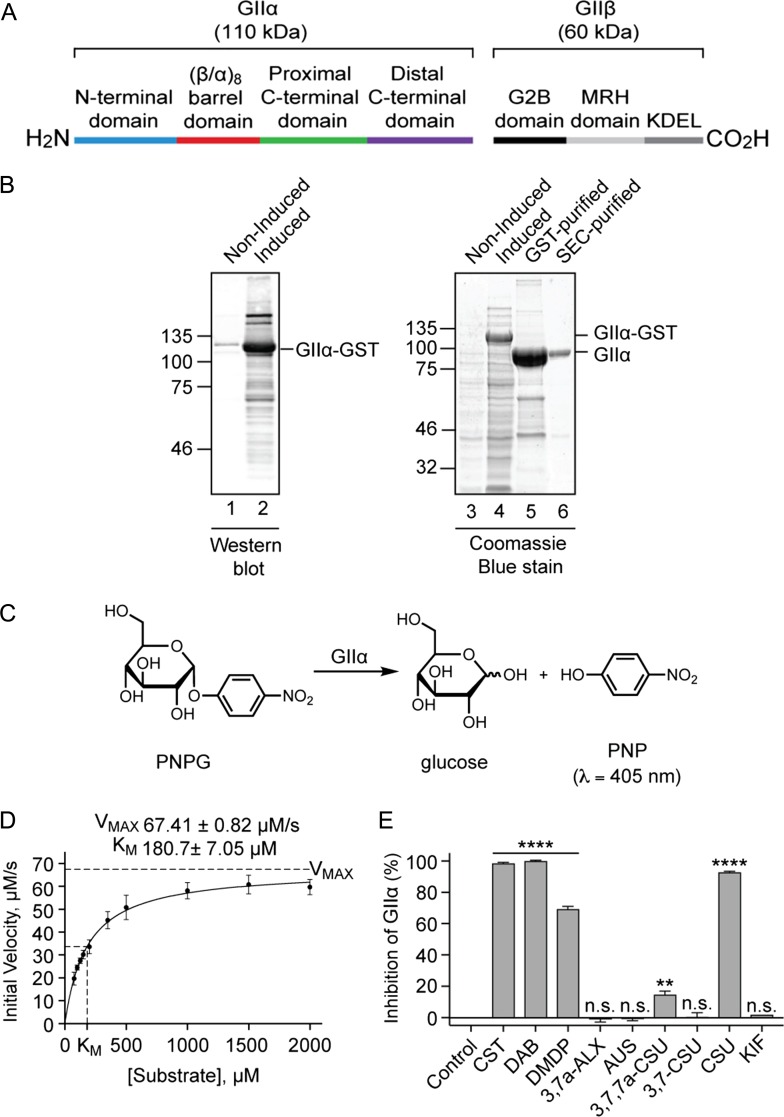
The effect of compounds on α-glucosidase II enzyme activity. (**A**) ER α-Glu II is a heterodimeric enzyme consisting of the catalytic GIIα subunit (110 kDa) and a non-catalytic regulatory GIIβ subunit (60 kDa). The active site of GIIα is located in its (β/α)_8_ barrel domain (Caputo et al. 2016; Satoh et al. 2016). The GIIβ subunit contains an N-terminal GIIα-binding domain (G2B), a mannose-6-phosphate receptor homology (MRH) domain involved in N-glycan recognition and a KDEL ER-retention signal (Olson et al. 2013; Caputo et al. 2016). (**B**) A C-terminally GST-tagged version of *Chaetomium thermophilum* GIIα lacking the signal sequence (Satoh et al. 2016) was expressed in *E. coli* (western blot using anti-GST, lanes 1–2; Coomassie Blue staining, lanes 3–4; see GIIα-GST). Following cell lysis, recombinant protein was purified using a Glutathione-Sepharose Column and GIIα released by on-column cleavage with tobacco etch virus (TEV) protease (lane 5), followed by size exclusion chromatography (SEC) (lane 6). (**C**) A schematic depicting the calorimetric reaction used to measure GIIα inhibition. GIIα-catalyzed cleavage of *p*-nitrophenyl-α-*D*-glucopyranoside (PNPG) produces glucose and yellow *p*-nitrophenol (PNP). The amount of PNP liberated during the course of the reaction was monitored by absorbance measurements. (**D**) Different concentrations of PNPG (ranging from 75 μM to 2 mM) were incubated with GIIα (6 μg/mL) at 37°C and absorbance measurements (λ = 410 nm, 1 min intervals, 90 min) used to generate a substrate-velocity curve. Using the Michaelis-Menten model, values for *V*_MAX_ (67.41 ± 0.82 μM/s) and *K*_M_ (180.7 ± 7.05 μM) were estimated (*n* = 12, *R*^2^ 0.9534). (**E**) Compounds at 100 μM were incubated with 125 μM PNPG and 6 μg/mL GIIα at 37°C and absorbance measurements (λ = 410 nm, 1 min intervals, 90 min) used to calculate the % inhibition of GIIα relative to control reactions. Assays were performed in triplicate (*n* = 3) and statistical significance (one-way ANOVA) determined using Tukey’s multiple comparisons test. Statistical significance is given as n.s., non-significant; **, *P* < 0.01 and ****, *P* < 0.0001.

Thus far, all our data suggest that the effects of the five active compounds observed during the cell-free translation of glycoproteins (CST, DAB, DMDP, 3,7a-ALX and CSU) are due to inhibition of glucose trimming via α-Glu I and/or α-Glu II, both of which are active in the ER lumen (cf. Figure 1). In order to better understand the molecular basis for the effects observed using ER microsomes, we sought to study the activity of the same compounds on the isolated catalytic alpha-subunit of the α-Glu II complex (GIIα) (Figure 5A).

Taking advantage of recent structural studies (Satoh et al. 2016), we expressed and purified the GIIα subunit from the thermophilic fungus *Chaetomium thermophilum* (Figures 5A–B, Supplementary Table SI) for use in a simple enzyme assay whereby the regulatory β subunit (GIIβ), and its role in N-glycan recognition (Olson et al. 2013), is not required for *in vitro* catalytic activity (Chapdelaine et al. 1978; Trombetta et al. 2001). Using *para*-nitrophenyl-α-*D*-glucopyranoside (PNPG) as a colorimetric substrate (Figure 5C), we estimated values for *V*_MAX_ of 67.4 ± 0.8 μM/s and *K*_M_ of 181 ± 7 μM for *C. thermophilum* GIIα (Figure 5D).

Inclusion of each of our panel of iminosugars (Figure 2) during the enzyme-catalyzed reaction showed that CST, DAB and CSU (Figures 2A, C and E) all strongly inhibited GIIα activity (Figure 5E). In contrast, DMDP (Figure 2D) was a moderate inhibitor and 3,7,7a-CSU (Figure 2H) a very weak inhibitor (Figure 5E). The compounds KIF, 3,7a-ALX, AUS and 3,7-CSU (Figures 2B, F, G and I) all had no significant effect on enzyme activity (Figure 5E). Of particular interest, is the inactivity of 3,7a-ALX (Figure 2F) towards GIIα since, in the cell-free system, the same compound was effective at inhibiting the ER processing of N-glycans (Figure 4D). As glucose trimming in ER microsomes involves both α-Glu I and α-Glu II, these data suggest that 3,7a-ALX may inhibit glucose trimming via its actions on α-Glu I rather than α-Glu II. This model is also consistent with the glycoprotein profiles of the Op91-2Gly products obtained using 3,7a-ALX and CST (see Figure 3D).

The mode of inhibition of CST, DAB, DMDP and CSU towards GIIα activity was determined using Lineweaver-Burk plots and all four compounds exemplified competitive inhibition (Supplementary Figure S3). Subsequently, *K*_i_ values for each compound were calculated from substrate-velocity curves using the Michaelis-Menten model for competitive inhibition (Supplementary Figure S4). Based on these calculations (Table I), we found that DAB is a very effective inhibitor of *C. thermophilum* GIIα (10-fold better than CST), CSU is comparable to CST, whilst DMDP is the weakest inhibitor.

**Table I.** Inhibition data of four active compounds towards ER α-Glu II as determined by a calorimetric kinetic assay using purified GIIα

Compound	Inhibition Mode	*K*_*i*_ (μM)^a^	*K*_*i*_ (μM)^b^
DAB	Competitive	0.187 ± 0.003	0.170 ± 0.003
CST	Competitive	2.60 ± 0.58	3.15 ± 0.10
CSU	Competitive	4.07 ± 0.06	4.15 ± 0.056
DMDP	Competitive	18.07 ± 0.51	14.95 ± 0.60

^a^*K*_*i*_ was calculated using *K*_M_ and *V*_MAX_ values obtained from controls (*n* = 3) per compound

^b^*K*_*i*_ was calculated using *K*_M_ and *V*_MAX_ values obtained from controls (*n* = 12) across all four compounds

### Inhibitors display different interactions with the GIIα active site *in silico*

In order to better understand the differences in the inhibitory activities of DAB, CST, DMDP and CSU, we next modeled these compounds into the substrate binding site of GIIα. To date, two high resolution structures of GIIα have been resolved, one utilizing *C. thermophilum* GIIα (Satoh et al. 2016) and the other a murine protein (Caputo et al. 2016). These proteins share 41% and 92% sequence identity respectively with the canine GIIα present in the ER microsomes used in our gel shift assays (cf. Figures 3 and 4), and both studies locate the putative enzyme active site in the center of a highly conserved (β/α)_8_ barrel domain (Supplementary Figure S5). The murine and *C. thermophilum* GIIα proteins have a similar domain architecture and the respective positioning of bound disaccharides is well matched (Caputo et al. 2018). Given its close similarity to both the canine (92% sequence identity) and human (90% sequence identity) proteins, we utilized the mouse GIIα structure in our docking studies in the hope of obtaining information of potential therapeutic value.

**Figure 6.**
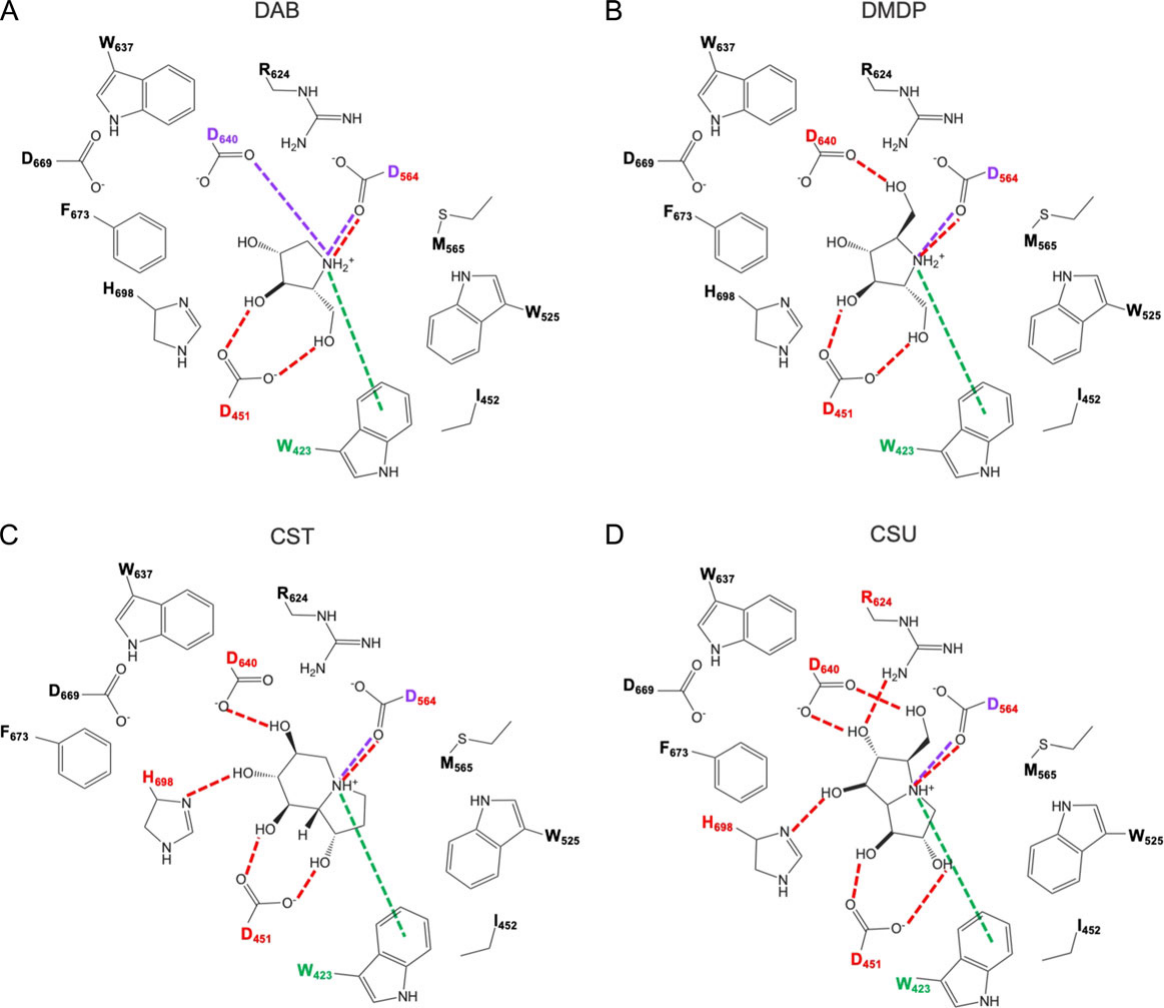
Inhibitors of ER α-Glu II exhibit similar binding modes but form different bonding interactions with GIIα when docked in the substrate binding site. (**A**) DAB, (**B**) DMDP, (**C**) CST and (**D**) CSU share common hydrogen bonding interactions (red dashed line) with D451 and D564, cation-π interactions (green dashed line) with W423 and an ionic interaction (purple dashed line) with D564. DAB may form a second ionic interaction with D640 not present in the other three compounds whose variable bonding network involves additional hydrogen bonding interactions with residues D460 and/or H698 and/or R624.

Our modeling studies led us to two clear conclusions; firstly, whilst all four of the compounds that we subjected to a detailed kinetic analysis may form an ionic bond with D564, only the most effective competitive inhibitor, DAB (see Table I), can potentially form a second ionic bond via D640 of the GIIα active site (see Figures 6A–D and Supplementary Figure S6); secondly, CST and CSU may both form a hydrogen bond with GIIα via H698, which is not formed by DMDP, the least effective inhibitor tested (Table I and Figures 6C–D versus Figure 6B). Residues D564, D640 and H698 are all conserved between the *C. thermophilum*, canine, murine and human ER α-Glu II enzymes (Supplementary Figure S5, see asterisks and filled circle). We, thus, conclude that the greater inhibitory potency of DAB is driven by an additional ionic interaction formed between the endocyclic nitrogen and GIIα whereas, for the other three compounds, their effectiveness as inhibitors most likely results from small differences in their binding affinity that are driven by their respective hydrogen bonding networks.

## Discussion

Here, we have evaluated nine iminosugars as inhibitors of the glycoprotein processing enzymes ER α-Glu I and ER α-Glu II using a combination of two *in vitro* approaches. Firstly, we analyzed the effects of compounds on the relative mobility of newly synthesized glycoproteins in the presence of ER microsomes and, secondly, we studied their ability to inhibit purified recombinant GIIα.

ER derived microsomes faithfully recapitulate protein N-glycosylation and subsequent glucose trimming events that occur in the ER lumen but we were unable to detect any evidence of mannose trimming and the commercial cell-free translation system we used precludes any ERAD of the model glycoproteins studied (Carlson et al. 2005; Vembar and Brodsky 2008). Our studies using ER microsomes, thus, allow us to draw two general conclusions in relation to the effects on glucose trimming that we observed with the compounds studied: (i) inhibition appears to be independent of the substrate that bears the N-linked glycan(s), the number of glycans present and the location/context of the glycan(s) within the polypeptide; (ii) their inhibitory effects are comparable whether N-linked glycans are added co-translationally or post-translationally, most likely via distinct mammalian OST complexes (Ruiz-Canada et al. 2009). On this basis, we conclude that five compounds (CST, DAB, DMDP, CSU and 3,7a-ALX, see Figure 2) inhibit overall glucose trimming in ER derived microsomes. Interestingly, although studies using purified microsomal enzyme fractions suggested AUS is a selective ER α-Glu II inhibitor (Tropea et al. 1989), it resulted in barely detectable levels of inhibition in our cell-free system (Figures 3C, 4C–D and Supplementary Figure S1). Hence, such gel shift assays may provide a useful *in vitro* tool in the search for broad spectrum antivirals by helping to identify new compounds that target host ER α-glucosidases (Chang, Block et al. 2013; Alonzi et al. 2017).

Complementary to our studies using ER microsomes, and in an attempt to distinguish between inhibitors of ER α-Glu I/II, we evaluated the same panel of iminosugars (Figure 2) for their ability to inhibit purified α-Glu II. To this end, we expressed and purified the catalytic GIIα subunit from *C. thermophilum* and used it in a simple enzyme assay performed in the presence and absence of the iminosugars. Our preliminary screen of compound activity revealed one very striking result, namely that 3,7a-ALX (Figure 2F) was completely inactive towards GIIα enzyme activity (Figure 5E), despite its effectiveness with ER microsomes (Figure 4D). The simplest explanation for these findings is that 3,7a-ALX is a selective inhibitor of ER α-Glu I and all our data are consistent with this hypothesis. Furthermore, the key residues implicated in substrate binding (Caputo et al. 2016, 2018; Satoh et al. 2016) are conserved between the *C. thermophilum* and canine alpha-subunits of ER α-Glu II (Supplementary Figure S5), suggesting that our data with the purified enzyme is directly relevant to our microsome based studies. Nevertheless, although we tentatively suggest that 3,7a-ALX may be a selective inhibitor of ER α-Glu I, further experiments, such as studies using purified ER α-Glu I, will be required to confirm this hypothesis.

The four compounds that did inhibit *C. thermophilum* GIIα in our preliminary screen (CST, DAB, DMDP, CSU, see Figure 5E) were subjected to a full kinetic analysis (Supplementary Figures S3–S4), confirming that they all act as competitive inhibitors and allowing us to rank order their effectiveness on the basis of their K_i_ values (Table I). In this purified enzyme assay, DAB was over 10-fold more effective than any of the three other compounds tested and we carried out *in silico* modeling of its interaction with the GIIα substrate binding site in order to better understand the structural basis for this enhanced inhibitory activity (Figure 6). Our modeling showed that only DAB has the potential to form a second ionic bond with residue D640 (the catalytic acid/base) of the GIIα active site (Figure 6 and Supplementary Figure S6). We propose that it is this additional ionic bond which enhances the inhibitory activity of DAB over the other three compounds analyzed (Supplementary Figure S6). Of the three other inhibitors, we find that CST and CSU are 4 to 7-fold more effective than DMDP, the least effective “active” compound that we tested (Table 1). In this case, our modeling suggests that this variation is based on differences in the number and/or strength of the hydrogen bonding interactions that these three compounds may form with GIIα. Hence, the effectiveness with which DMDP forms hydrogen bonds with the ER α-Glu II substrate binding site is most likely marginally lower than that for CST or CSU (cf. Figure 6).

Despite the differences in their potential bonding interactions within the GIIα active site, all four compounds exhibit *K*_i_ values in the mid to low μM range (Table I). Given that inhibition of ER α-Glu I is sufficient but not obligate for antiviral activity (Kiappes et al. 2018), our kinetic and *in silico* characterization of CST, DAB, DMDP and CSU as inhibitors of ER α-Glu II presents a potential platform for the development of therapeutic antivirals targeting host ER α-glucosidases. Interestingly, the most selective ER α-Glu II inhibitor to date, the recently identified DNJ-tocopherol conjugate (ToP-DNJ) (Kiappes et al. 2018), is a second-generation derivative of DNJ. Hence, incorporation of the tocopherol moiety into the iminosugar scaffold of CST, DAB, DMDP or CSU may yield an iminosugar-conjugate with increased selectivity towards ER α-Glu II (cf. Kiappes et al. 2018).

Furthermore, whilst antiviral activity resulting from inhibition of ER α-Glu I/II has been attributed to abrogation of glucose trimming and failure to enter and/or exit the CNX-CRT cycle, it is also conceivable that, through inhibition of ER α-Glu II, glycoproteins may participate in an alternative, and mechanistically distinct, protein quality control pathway involving the carbohydrate binding protein malectin (Figure 1C), which specifically associates with di-glucosylated N-glycans (Schallus et al. 2008; Galli et al. 2011). Malectin preferentially binds misfolded ERAD substrates as opposed to partially/correctly folded glycoproteins (Chen et al. 2011). However, although the malectin-glycoprotein association is G2M9-dependent, a G2M9 N-glycan alone is not sufficient to selectively distinguish between glycoproteins based on their level of folding (Qin et al. 2012). Instead, it is proposed that a complex formed by malectin and the ribophorin I subunit of the OST (cf. Figure 1) exerts glycoprotein quality control for these substrates (Quin et al. 2012, Yang et al. 2018). Thus, by selectively inhibiting ER α-Glu II the resulting accumulation of di-glucosylated N-glycan species may lead to an increased association with the malectin-ribophorin I complex as well as a stalling of the CNX-CRT cycle, thereby disrupting viral glycoprotein folding through two mechanistically distinct quality control pathways.

In summary, we have utilized a cell-free system which recapitulates N-glycosylation events in the ER, performed kinetic studies with purified GIIα and employed a docking model of the GIIα active site to characterize a panel iminosugars as inhibitors of the glycoprotein processing enzymes ER α-Glu I and ER α-Glu II. Our study extends the chemical space surrounding ER α-Glu I/II iminosugar inhibitors whereby we identify the C-3 substituted pyrrolizidines CSU and 3,7a-ALX as promising second-generation iminosugars.

## Materials and methods

### Compounds

CST (Figure 2A) and KIF (Figure 2B) were purchased from Generon Ltd and Sigma respectively. Compounds purified from plant sources were as previously described: DAB (Figure 2C) (Nash et al. 1985), DMDP (Figure 2D) (Kato et al. 1999), CSU (Figure 2E) (Nash et al. 1994), 3,7a-ALX (Figure 2F) (Nash et al. 1988) and AUS (Figure 2G) (Kato et al. 2003). 3,7-CSU (Figure 2I) was synthesized as previously described (Bell et al. 1997) and 3,7,7a-CSU (Figure 2H) was isolated by PhytoQuest Ltd as a by-product from the synthesis of 3,7-CSU.

### Cell-free analysis of ER α-glycosidase inhibition

Linear DNA templates were generated by PCR and transcribed into RNA using T7 or SP6 polymerase (Promega) as previously described (Crawshaw 2004, Johnson et al. 2012; Watson et al. 2013). OPG1Kcv was generated by site-directed mutagenesis of OPG2Kcv in which residue T4 was mutated to A. Translation reactions (20 μL) were performed in nuclease-treated rabbit reticulocyte lysate (Promega) in the presence of EasyTag EXPRESS ^35^S Protein Labelling Mix containing [^35^S] methionine (Perkin Elmer) (0.533 MBq; 30.15 TBq/mmol), ~2.5% (*v/v*) amino acids minus methionine (Promega) and ~10% of *in vitro* transcribed mRNA encoding OPG1Kcv (360 ng/μL stock), OPG2Kcv (330 ng/μL stock), Op91 (450 ng/μL stock), ppcecAOPG1 (340 ng/μL stock), ppcecAOPG2 (150 ng/μL stock) or ppαF (330 ng/μL stock). For co-translational reactions, 6.5% (*v/v*) nuclease-treated ER microsomes (from stock with OD_280_ = 44/mL) were added and samples were incubated for 20 min at 30°C. In the post-translational system, protein synthesis was performed in the absence of ER microsomes for 20 min at 30°C. Following translation, puromycin was added to 0.1 mM and samples incubated for 10 min at 30°C to ensure translational termination and ribosome release. 10% (*v*/*v*) ER microsomes were then added and samples incubated for 20 min at 30°C to enable membrane translocation. 5% (*v*/*v*) compound (5 mM final concentration, from 100 mM stock solutions in H_2_O), or an equal volume of H_2_O, were added at the same time as ER microsomes in both co- and post-translational systems. Membrane-associated fractions were isolated by centrifugation through an 80 μL high-salt cushion (0.75 M sucrose, 0.5 M KOAc, 5 mM Mg(OAc)_2_, 50 mM Hepes-KOH, pH 7.9) at 100,000 ***g*** for 10 min at 4°C and taken directly in SDS sample buffer (0.02% bromophenol blue, 62.5 mM, 4% (*w*/*v*) SDS, 10% (*v*/*v*) glycerol, pH 7.6, 1 M dithiothreitol). Where indicated, samples were treated with 1000 U of Endo (New England Biolabs) for 1 h at 37°C prior to SDS-PAGE analysis. Radiolabelled products were visualized using a Typhoon FLA-7000 (GE Healthcare) following exposure to a phosphorimaging plate for 24–72 h. For ppcecAOPG2, quantitative analysis of glucose trimming inhibition was performed using AIDA v.5.0 (Raytest Isotopenmeβgeräte) whereby the intensity of 2Gly signals with reduced glycoprotein mobility was expressed relative to the 2Gly signal exhibiting the same level of migration as the no-inhibitor control.

### Expression and purification of GIIα

The parental vector, pCold-glutathione *S*-transferase (pCold-GST) (Takara Bio Inc.), was purchased from Clontech. *Chaetomium thermophilum* GIIα (residues 31–977) subcloned into pCold-GST was a gift from Tadashi Satoh and Koichi Kato (Nagoya City University, Japan) and the protein was purified as previously described (Satoh et al. 2016). Briefly, GIIα-GST was expressed in *E*. *coli* BL21-CodonPlus (DE3, Agilent Technologies) according to the manufacturer (Takara Bio Inc.) and the GST-fused protein captured on a Glutathione-Sepharose column (GE Healthcare). Incubation with tobacco etch virus (TEV) protease released GIIα which was further purified by size exclusion chromatography (SEC) on a Superose 6, 10/3000 GL Column (GE Healthcare) run in PBS. Samples taken during expression and purification were analyzed by Coomassie Blue staining, western blotting and mass spectrometry as indicated.

### SDS-PAGE and western blotting

All samples were suspended in SDS sample buffer and denatured for 10 min at 70°C prior to resolution by SDS-PAGE (120 V, 120 min). Translation reactions (16% PAGE) were fixed for 5 min (20% MeOH, 10% AcOH), dried for 2 h at 65°C and products detected by phosphorimaging. GIIα samples (10% PAGE) were either stained with InstantBlue Coomassie Protein Stain (Expedeon) for 1 h and de-stained in H_2_O for 16 h or transferred to a PVDF membrane in transfer buffer (0.06 M Tris, 0.60 M glycine, 20% MeOH) at 300 mA for 2.5 h for western blot analysis. After transfer, PVDF membranes were incubated in Casein blocking buffer (Sigma) made up in TBS, incubated with primary antibody (anti-GST (1:1,000), GE Healthcare, 27-4577-01) and processed for fluorescence-based detection as described by LI-COR Biosciences (Secondary antibody (1:10,000) IRDye 680 RD Donkey anti-Goat IgG). Signals were visualized using an Odyssey CLx Imaging System (LI-COR Biosciences).

### Mass spectrometry

Purified GIIα was subjected to SDS-PAGE, Coomassie protein staining and the band of interest (~100 kDa) was excised from the gel and dehydrated using acetonitrile and vacuum centrifugation. Dried gel pieces were reduced with 10 mM dithiothreitol, alkylated with 55 mM iodoacetamide and gel pieces were then washed alternately with 25 mM ammonium bicarbonate followed by acetonitrile. This was repeated, and the gel pieces dried by vacuum centrifugation prior to digestion with trypsin for 16 h at 37°C. Digested samples were analyzed by LC-MS/MS using an UltiMate^®^ 3000 Rapid Separation LC (RSLC, Dionex Corporation, Sunnyvale, CA) coupled to an Orbitrap Elite (Thermo Fisher Scientific, Waltham, MA) mass spectrometer. Peptide mixtures were separated using a gradient from 92% A (0.1% FA in water) and 8% B (0.1% FA in acetonitrile) to 33% B, in 44 min at 300 nL min^−1^, using a 75 mm × 250 μm i.d. 1.7 mM BEH C18, analytical column (Waters). Peptides were selected for fragmentation automatically by data dependent analysis. Data produced were searched using Mascot (Matrix Science UK), against the amino acid sequence of GIIα (Supplementary Figure S5) and the *Swi*ssprot and Trembl database with taxonomy of *E. coli* selected. Data were validated using Scaffold (Proteome Software, Portland, OR).

### Enzyme kinetics of purified recombinant GIIα

Inhibition of GIIα was determined by measuring the amount of PNP (yellow) released from PNPG in the absence and presence of each compound. All absorbance (*λ* = 410 nm) and pathlength measurements were acquired from 180 μL samples (pH 7.4, 37°C) in black 96-well microtitre plates (Falcon, product 353219) using a Synergy H1 Hybrid multi-mode plate reader (BioTek). PNPG (from 22.5 mM–675 μM stock solutions in PBS), compound (from 0.9 mM–0.88 μM stock solutions in H_2_O) and GIIα (6 μg/mL in PBS per reaction) each constituted 9% (*v/v*) of total sample volume in PBS. Each experiment included control samples in which blank (PBS and H_2_O), PNP only, PNPG only, compound only and GIIα only samples were present at the same time as 1 enzyme-substrate (no inhibitor control) and 11 enzyme-compound-substrate samples with concentrations provided in the appropriate figure legend. The kinetic protocol consisted of plate insertion, incubation for 5 min, plate shaking for 1 min, plate ejection for 30 s during addition of GIIα using a 12-channel pipette, plate insertion, plate shaking for 10 s and measurement of absorbance values at 1 min intervals for 90 min. Absorbance measurements were converted into the concentration of liberated PNP (Molar Extinction Coefficient, *ε* = 18.5 mM^−1^ cm^−1^) using the Beer–Lambert law and, subsequently, into initial velocity, νο. Inhibition of GIIα was determined by:

$\%\;\mathrm{Inhibition}=\;\left(\frac{\nu ο\left(\mathrm{control}\right)-\;\nu ο\left(\mathrm{compound}\right)}{\nu ο\left(\mathrm{control}\right)}\right)\;\times \;100$

All regression analysis was performed using Prism 7.0d (GraphPad). Non-linear regression analysis was used to calculate *V*_MAX_ and *K*_M_ values and generate Lineweaver–Burk plots using the Michaelis–Menten and Briggs–Haldane kinetic model and *K*_i_ values were determined using the Michaelis–Menten model for competitive inhibition.

### *In silico* docking analyses

Six crystal structures of murine ER α-Glu II were obtained from the Protein Data Bank (PDB ID: 5H9O, 5HJO, 5HJR, 5IED, 5IEE and 5IEF), and docking calculations conducted using the ensemble docking protocol in order to consider structural variations of the protein upon the ligand binding. The protein structure was performed using Maestro Protein Preparation Wizard (version 11.6, Schrödinger, LLC, New York, NY, USA). Two water molecules were consistently observed inside the ligand-binding sites of the six crystal structures, thus, we considered these water molecules in the docking process and removed any other water molecules from the crystal structure. The docking site was defined as an enclosing box (10 × 10 × 10 Å) centered at the centroid of the co-crystallized ligand. The three-dimensional structures of the ligands were built using Chem3D (PerkinElmer, Inc., Waltham, MA, USA), hydrogens were added at neutral pH 7.4 using LigPrep (Schrödinger, LLC, New York, NY, USA), and then, multiple conformations were generated using ConfGen (Schrödinger, LLC, New York, NY, USA). LigPrep can produce multiple structures with various ionization states and ring conformations from the input structure. In the first instance, the docking analyses were performed under SP (standard precision) mode using the six protein structures with Glide (version 7.9, Schrödinger, LLC, New York, NY, USA) and then the structure of the ligand bound complex exhibiting the best Glide SP were selected for each protein. Next, the rigid docking analyses were performed under XP (extra precision) mode using these pre-selected complex structures. Finally, the complex structures exhibiting the best Glide XP score were selected and energy minimized using MacroModel (version 12.0, Schrödinger, LLC, New York, NY, USA) with an OPLS3e force field and 0.05 kcal/mol/Å of convergence with a distance-dependent dielectric constant (*ε* = 4). The validity of this docking strategy was proven by cross docking analyses using six different co-crystallized ligands. All calculations were performed using the Schrödinger Suite 2018-2 (Schrödinger, LLC, New York).

### Statistical analysis

Statistical analyses (ANOVA) were performed using Prism 7.0d (GraphPad) and defined in figure legends with the type of multiple comparisons test used, number of technical replicates (*n*) and goodness of fit (*R*^2^) as appropriate. Statistical significance is given as n.s., non-significant; *, *P* < 0.05; **, *P* < 0.01; ***, *P* < 0.001 and ****, *P* < 0.0001.

## Supplementary Material

Supplementary Data

Supplementary Data

## Abbreviations

AUS: australine
CNX: calnexin
CRT: calreticulin
CST: castanospermine
CSU: casuarina
DAB: 1,4-dideoxy-1,4-imino-*D*-arabinitol
DNJ: 1-deoxynojirimycin
DMDP: 2,5-dideoxy-2,5-imino-*D*-mannitol
Endo H: endoglycosidase H
ER: endoplasmic reticulum
ERAD: ER-associated degradation
GST: glutathione *S*-transferase
GIIα: alpha-subunit of alpha-glucosidase II
GIIβ: beta-subunit of alpha-glucosidase II
*K*_i_: inhibitory constant
KIF: kifunensine
*K*_M_: Michaelis constant
MRH: mannose-6-phosphate receptor homology
OPG1 epitope: residues 1–26 or a 14 residue N-terminal fragment of bovine rhodopsin containing one N-glycosylation site
OPG2 epitope: residues 1–18 or 1–26 of bovine rhodopsin containing two N-glycosylation sites
OPG1Kcv: OPG2Kcv in which one N-glycosylation site was removed by site-directed mutagenesis of T4A
OPG2Kcv: potassium channel protein Kcv with an OPG2 epitope engineered at the N-terminus
Op91: residues 1–91 of bovine rhodopsin
OST: oligosaccharyltransferase
PBS: Dulbecco’s phosphate buffered saline
PNP: *para*-nitrophenol
PNPG: para-nitrophenyl α-*D*-glucopyranoside
ppcecAOPG2: preprocecropin A with an OPG2 epitope engineered at the C-terminus
ppαF: prepro-alpha-factor
SDS-PAGE: sodium dodecyl sulfate polyacrylamide electrophoresis
SEC: size exclusion chromatography
TBS: Tris buffered saline
TEV: tobacco etch virus
ToP-DNJ: DNJ-tocopherol conjugate
UGGT: UDP-glucose:glycoprotein glucosyltransferase
*V*_MAX_: maximum velocity
1Gly: singly N-glycosylated protein
2Gly: doubly N-glycosylated protein
3,7-CSU: 3,7-di*epi*-casuarine
3,7a-ALX: 3,7a-di*epi*-alexine
3,7a-CSU: 3,7a-di*epi*-casuarine
α-Glu I: alpha-glucosidase I
α-Glu II: alpha-glucosidase II
