# Correction to: Characterizing the selectivity of ER α-glucosidase inhibitors

**DOI:** 10.1093/glycob/cwad017

**Published:** 2023-03-25

This is a correction to: Sarah O'Keefe, Quentin P Roebuck, Izumi Nakagome, Shuichi Hirono, Atsushi Kato, Robert Nash, Stephen High, Characterizing the selectivity of ER a-glucosidase inhibitors, *Glycobiology*, Volume 29, Issue 7, July 2019, Pages 530–542, https://doi.org/10.1093/glycob/cwz029

In the originally published version of this manuscript, the supplementary data file was incorrect. It showed changes to the text that the authors made in response to the reviewers' comments.

The correct supplementary data:

Supplementary Data 13.3.19.pdf

These details have been corrected only in this correction notice to preserve the published version of record.

## Supplementary Material

Supplementary_Data_13_3_19_cwad017

