# Supplementary material for: Characterizing the selectivity of ER α-glucosidase inhibitors
Source: Glycobiology. 2019 Apr 29;29(7):530–42. doi: 10.1093/glycob/cwz029 (PMC6583763; doi:10.1093/glycob/cwz029)
Supplement: Supplementary Data [file cwz029_for_reviewers_only_highlighted_text_changes,_14.3.19.pdf]

Characterising the selectivity of ER α-glucosidase inhibitors

Keywords: endoplasmic reticulum/iminosugar inhibitors/glucose trimming/N-linked glycosylation

Sarah O'Keefe[1], Quentin P. Roebuck[1], Izumi Nakagome[2], Shuichi Hirono[2], Atsushi Kato[3], Robert Nash[4], Stephen High[5]

[1] School of Biological Sciences, Faculty of Biology, Medicine and Health, University of Manchester, Manchester, M13 9PT, United Kingdom.

[2] School of Pharmaceutical Sciences, Kitasato University, Tokyo 108-8641, Japan.

[3] Department of Hospital Pharmacy, University of Toyama, 2630 Sugitani, Toyama 930-0194, Japan.

[4] PhytoQuest Ltd, Plas Gogerddan, Aberystwyth, Ceredigion, SY23 3EB, United Kingdom.

[5] To whom correspondence should be addressed: Tel: ++44 161 275 5070; Fax ++44 275 5082; e-mail: stephen.high@manchester.ac.uk

Running Head: Inhibiting glucose trimming in the ER

Supplementary Data Included: Figures S1-S6 and Table SI

Figure S1. *The effects of active compounds are independent of glycoprotein substrate*

Figure S2. *Migration profiles of glycoprotein species generated by active compounds*

Figure S3. *Active compounds are competitive inhibitors of ER α-glucosidase II*

Figure S4. *K_i calculations for ER α-glucosidase II inhibitors*

Figure S5. *Alignment of ER α-glucosidase II α-subunit sequences*

Figure S6. *Bonding between active inhibitors and residue D_{640} of mouse GIIα*

Table SI. *Mass spectrometric analysis of GIIα (C. thermophilum)*

Abstract

The endoplasmic reticulum (ER) contains both α-glucosidases and α-mannosidases which process the N-linked oligosaccharides of newly synthesised glycoproteins and thereby facilitate polypeptide folding and glycoprotein quality control. By acting as structural mimetics, iminosugars can selectively inhibit these ER localised α-glycosidases, preventing N-glycan trimming and providing a molecular basis for their therapeutic applications. In this study, we investigate the effects of a panel of nine iminosugars on the actions of ER luminal α-glucosidase I and α-glucosidase II. Using ER microsomes to recapitulate authentic protein N-glycosylation and oligosaccharide processing, we identify five iminosugars that selectively inhibit N-glycan trimming. Comparison of their inhibitory activities in ER microsomes against their effects on purified ER α-glucosidase II, suggests that 3,7a-di*epi*-alexine acts as a selective inhibitor of ER α-glucosidase I. The other active iminosugars all inhibit α-glucosidase II and, having identified 1,4-dideoxy-1,4-imino-*D*-arabinitol (DAB) as the most effective of these compounds, we use *in silico* modelling to understand the molecular basis for this enhanced activity. Taken together, our work identifies the C-3 substituted pyrrolizidines casuarine and 3,7a-di*epi*-alexine as promising "second-generation" iminosugar inhibitors.

Introduction

Widely distributed in plants, iminosugars represent a structurally diverse group of compounds, comprised of both monocyclic (piperidines and pyrrolidines) and bicyclic scaffolds (indolizines, pyrrolizidines and *nor*tropanes); and their isolation from natural sources, chemical syntheses and biological evaluation are the subject of several comprehensive reviews (Stütz 1999, Asano 2008, Asano et al. 2000, Watson et al. 2001, Compain and Martin 2007). Due to their structural mimicry of natural substrates, iminosugars have therapeutic potential in several areas of disease and have been evaluated for inhibitory activity towards a variety of α- and β-glycosidases (de Melo et al. 2006, Compain et al. 2007).

Amongst these enzymes, α-glucosidase I (α-Glu I, EC 3.2.1.106) (Barker and Rose 2013) and α-glucosidase II (α-Glu II, EC 3.2.1.84) (Satoh et al. 2016, Caputo et al. 2016) are resident within the lumen of the endoplasmic reticulum (ER), where they process newly synthesised glycoproteins. This processing involves the sequential removal, or 'trimming', of glucose residues from the G3M9 N-glycan moiety initially added to newly synthesised glycoproteins either during or after protein synthesis (Figure 1). Trimming to G2M9 facilitates binding to malectin (Tannous et al. 2015), whilst further trimming to G1M9 enables binding to calnexin (CNX) and calreticulin (CRT) (Helenius and Aebi 2004, Tannous et al. 2015). CNX and CRT, together with the oxidoreductase ERp57, promote the correct folding and maturation of G1M9 containing glycoprotein substrates (Oliver et al. 1997, Tannous et al. 2015). Following dissociation from CNX/CRT, removal of the final glucose residue by ER α-Glu II and removal of a mannose residue by ER α-mannosidase I (ER Man I) enables the onward transport of properly folded glycoproteins to the Golgi complex where N-

linked glycans may be further remodelled (Figure 1; see also Helenius and Aebi 2004, Tannous et al. 2015). In the case of partially folded/misfolded glycoproteins, selective reglucosylation by the folding sensor UDP-Glc:glycoprotein glucosyltransferase (UGGT, EC 2.4.1-) regenerates the mono-glucosylated N-glycan restoring their ability to rebind CNX/CRT. This constitutes a cycle of transient cleavage and re-addition of the innermost glucose residue (the CNX-CRT cycle) in which CNX/CRT, UGGT and ER α-Glu II work in concert to assist protein folding in the ER lumen (Figure 1). Glycoproteins that are unable to reach a native conformation are subject to sequential mannose trimming steps via ER Man I and the ER degradation-enhancing mannosidase-like proteins (EDEMs) which direct these terminally misfolded glycoproteins into a pathway(s) for ER associated degradation (ERAD) (Helenius and Aebi 2004, Ninagawa et al. 2014, Tannous et al. 2015, Słomińska-Wojewódzka and Sandvig 2015), thereby preventing their progress through the secretory pathway.

Inhibition of ER α-Glu I and ER α-Glu II precludes entry into, and/or exit from, the CNX-CRT cycle by stalling glycoproteins in an either untrimmed (G3M9) or partially trimmed (G2M9/G1M9) forms. In each case, such intermediates offer scope to enhance our understanding of glycoprotein quality control processes within the ER (Araki and Nagata 2011, Tannous et al. 2015). ER α-Glu I/II inhibitors also have potential as antiviral agents which, given the paucity of broad-spectrum antivirals, warrants further investigation (Bekerman and Einav 2015). Indeed, the ER α-glucosidase inhibitors 1-deoxynojirimycin (DNJ), castanospermine (CST, Figure 2A), and their derivatives, exhibit antiviral activity towards many enveloped viruses, including: HIV (Gruters et al. 1987, Walker et al. 1987, Fleet et al. 1988); Dengue

(Whitby et al. 2005, Miller et al. 2012, Watanabe et al. 2016) and Ebola virus (Chang et al. 2013a). It is postulated that the abrogation of glucose trimming of viral glycoproteins, via inhibition of host ER α-Glu I/II, is sufficient to inhibit virion assembly and secretion (Chang et al. 2013b, Alonzi et al. 2017). However, the clinical development of DNJ, CST and their analogues has been impeded by modest reductions in viraemia and/or lack of clinical benefit (Miller et al. 2016, Ma et al. 2018, Low et al. 2014, Warfield et al. 2017). Strategies to improve the therapeutic efficacy, potency and tolerability of these compounds are ongoing (Ouzounov et al. 2002, Woodhouse et al. 2008, Watanabe et al. 2016, Ma et al. 2017, Ma et al. 2018, Kiappes et al. 2018).

Whilst CST, DNJ and their analogues have been studied in detail, as a bioactive class, iminosugars remain an underexplored area of chemical space (Horne et al. 2010) from which more potent inhibitors of ER α-Glu I/II may emerge. To this end, we sought to evaluate a subclass of polyhydroxylated pyrrolizidines bearing a C-3 substituent (Figures 2E-2I) as ER α-Glu I/II inhibitors and we report their effects on the ER processing of N-linked glycans in a cell-free translation system supplemented with ER-derived microsomes. These compounds are compared to the well characterised α-glycosidase inhibitors CST (dual ER α-Glu I/II), (Pan et al. 1983, Kaushal et al. 1988),1,4-dideoxy-1,4-imino-*D*-arabinitol (DAB, ER α-Glu II) (Asano et al. 1994) and 2,5-dideoxy-2,5-imino-*D*-mannitol (DMDP, ER α-Glu I) (Elbein et al. 1984, Asano et al. 1994) (Figures 2A, 2C and 2D) together with the mannosidase inhibitor kifunensine (KIF, ER Man I, Figure 2B) (Elbein et al. 1990). To complement these studies, we analysed the ability of the same panel of iminosugars to inhibit the enzyme activity of purified recombinant ER α-Glu-II, determined the inhibitory

constants (K_i) for the four most potent inhibitors and used *in silico* modelling to establish structure-activity-relationships for these compounds.

Results

A subset of iminosugars alter the ER processing of N-linked glycans

The effects of nine compounds (Figure 2) on the ER processing of N-linked glycans were examined using a cell-free system in which radiolabelled precursor proteins are synthesised in the presence of canine pancreatic microsomes analogous to the ER (Blobel and Dobberstein 1975, Pool and Dobberstein 2011). This system faithfully recapitulates the co-translational translocation of nascent polypeptides into, and across, the ER membrane and exposes them to the N-glycosylation machinery located in the ER lumen (Walter and Blobel 1983). Suitable asparagine residues (Mononen and Karjalainen 1984, Gavel and von Heijne 1990) in the nascent polypeptide chain are covalently modified with the high mannose form of the N-glycan and these glycans then rapidly undergo initial trimming reactions characteristic of the ER (Figure 1) (Helenius and Aebi 2004). Following glycoprotein synthesis, the ER membranes were recovered by centrifugation and associated radiolabelled proteins were resolved by SDS-PAGE and visualised by phosphorimaging (Figure 3A).

In order to maximise the effect of inhibiting N-glycan trimming as assessed by changes in mobility on SDS-PAGE, we initially studied a small polypeptide with multiple N-linked glycans. To this end, the previously characterised N-terminal fragment of bovine rhodopsin (Op91) (Crawshaw et al. 2004) containing two endogenous N-glycosylation sites (hereafter denoted the OPG2 epitope) was used as a model substrate for co-translational translocation (Figure 3B). The major non-glycosylated (0Gly) and doubly N-glycosylated (2Gly) species of the Op91 polypeptide synthesised in the presence of ER-derived microsomes were identified

by treatment with endoglycosidase H (Endo H) (EC 3.2.1.96), which resulted in the loss of N-glycosylated species (Figure 3C, lanes 1 and 2). Inclusion of the commercially available α-Glu I/II inhibitor CST during translation (cf. Oliver et al. 1997) resulted in a clear reduction in the mobility of the predominant 2Gly form of the Op91 polypeptide in comparison to the non-inhibitor control (Figure 3C, lanes 1 and 3). In contrast, the ER Man I inhibitor KIF (Elbein et al. 1990) had no obvious effect on the mobility of N-glycosylated species when compared to the control (Figure 3C, lanes 1 and 11). Hence, we conclude that alterations in Op91-2Gly mobility can be used to report an inhibition of glucose trimming *in vitro*.

Of the seven other compounds tested, DAB (Figure 2C), DMDP (Figure 2D) and casuarine (CSU, Figure 2E) resulted in a reduction of glycoprotein mobility that appeared comparable to CST (Figure 2A) whilst 3,7a-di*epi*-alexine (3,7a-ALX, Figure 2F) resulted in a doublet of products with reduced mobility (Figure 3C, lanes 1, 3, 4, 5, 6 and 10). The signal intensity profile of the products obtained in the presence of CST and the absence of any inhibitor provided benchmarks for the unprocessed G3M9 (Figure 3D, CST, see asterisk) and processed N-linked glycoproteins (Figure 3D, control) respectively. Profiles of the doubly N-glycosylated species of Op91 confirmed that DAB, DMDP, 3,7a-ALX and CSU also lead to a reduction in glycoprotein mobility, albeit to a varying degree (Figure 3D, see ΔGly). In contrast, any effect of 3,7,7a-tri*epi*-casuarine (3,7,7a-CSU, Figure 2H) on Op91-2Gly mobility was rather modest whilst australine (AUS, Figure 2G), and 3,7-di*epi*-casuarine (3,7-CSU, Figure 2I) had no obvious effect when compared to control and KIF treated products (Figure 3C, lanes 1, 7, 8, 9 and 11). Our finding that KIF has no obvious effect in our gel shift assay is consistent with previous reports that the inhibition of ER

dependent mannose trimming is difficult to detect via changes in glycoprotein mobility on SDS-PAGE (Cannon and Helenius, 1999). We therefore conclude that the alterations to the migration of Op91-2Gly that we observe in the presence of particular compounds (CST, DAB, DMDP, 3,7a-ALX and CSU) are most likely due to their inhibitory effects on glucose trimming via ER luminal α-Glu I and/or α-Glu II.

Op91-2Gly species contain two N-linked glycans making it difficult to attribute an inhibitor-dependent reduction in mobility to a precise N-glycan structure(s). However, when compared to the single major peak seen in the presence of 5 mM CST (presumed to be G3M9, see Figure 3D, CST, asterisk), a broader range of slightly faster migrating species was seen with both DAB and CSU (Figure 3D). We speculate that these products represent the accumulation of G2M9 and/or G1M9, forms of N-linked glycans consistent with the inhibition of ER α-Glu II (cf. Figure 1C). For DMDP and 3,7a-ALX the major glycoprotein species co-migrated with that of CST, although 3,7a-ALX treatment also resulted in a second prominent species of faster migration (Figure 3D). On this basis we propose that DMDP and 3,7a-ALX most likely both inhibit ER α-Glu I but do so less effectively than CST (cf. Figure 3D).

In order to establish whether the presence of multiple N-glycans influenced our ability to detect inhibitor-dependent changes in N-glycan trimming we repeated our experiments using model glycoproteins with a single N-glycosylation site (Supplementary Figure S1). In the case of the viral potassium channel Kcv, a version of the protein bearing exclusively one N-linked glycan (Supplementary Figure S1A, see 1Gly species) showed similar changes in mobility to those seen with both Op91-2Gly (Figure 3C) and OPG2Kcv (Watson et al.) a doubly N-glycosylated version of

Kcv (Supplementary Figures S1Aiii-iv, see 2Gly species). Thus, CST, DAB, DMDP, 3,7a-ALX and CSU all reduced the mobility of 1Gly and 2Gly species in a similar fashion. Likewise, the trends in the changes to glycoprotein mobility were comparable when singly and doubly N-glycosylated versions of the short secretory protein preprocecropinA (Johnson et al. 2012) were analysed by SDS-PAGE (Supplementary Figure S1B). However, subtle qualitative differences between the effects of active inhibitors were more apparent in the 2Gly form of the protein (Supplementary Figures S1Bii and S1Biv, cf. lanes 4 and 10). When migration profiles for these two additional doubly N-glycosylated glycoprotein substrates were analysed in comparison to CST, we again found that the effects of DAB and CSU were distinct from those of DMDP and 3,7a-ALX (Supplementary Figures S2A-S2B and Figure 3D). Taken together, these data suggest that the changes in glycoprotein mobility we observe are a valid reporter for the differences in the inhibitory actions of compounds with respect to ER α-Glu I and or ER α-Glu II.

Inhibition of N-glycan trimming is independent of when the glycan is added

Op91 provides a *bone fide* substrate for co-translational N-glycosylation (Figure 1A) since its N-terminal domain, which bears two N-glycosylation sites, is translocated into the ER lumen once the ribosome bound nascent chain arrives at the Sec61 translocon (Meacock et al. 2002). Since all of the model glycoproteins we have analysed to date all contained N-glycosylation sites derived from the N-terminus of Op91, we next investigated whether the location, or context, of the N-linked glycans added to glycoprotein substrates influences the ability of our chosen compounds to inhibit their ER processing. To this end, the yeast secretory glycoprotein prepro-alpha-factor (ppαF), bearing three naturally occurring N-linked glycans

(Supplementary Figure S1C) (Waters et al. 1988), was used as an additional model glycoprotein substrate. Band migration analysis showed that the same five compounds that altered the mobility of the previous three model substrates also resulted in the perturbation of ppαF-Gly migration (cf. Supplementary Figures S1C and S2C versus Figures 3C-3D), although the presence of three N-linked glycans did increase the complexity of the products (cf. Supplementary Figure S2C).

In addition to co-translational N-glycosylation, a subset of precursors and N-glycosylation sites can be modified post-translationally (Ruiz-Canada et al. 2009). We, therefore, exploited the ability of the short secretory protein preprocecropin A supplemented with a C-terminal OPG2 tag (ppcecAOPG2) to act as a well-defined post-translational substrate for N-glycosylation (Johnson et al. 2012). Following translation of ppcecAOPG2 mRNA, the separation of protein synthesis and N-glycosylation was achieved using puromycin to terminate protein synthesis prior to the addition of ER microsomes, thereby ensuring that membrane translocation proceeded via a post-translational, but Sec61-mediated, pathway (Figure 4A; see also Johnson et al. 2012, Zimmerman et al. 1990). In this way, the strictly post-translational N-glycosylation of the C-terminal tag of ppcecAOPG2 (Figure 4B) could be investigated. Strikingly, once again, the same five compounds (CST, DAB, DMDP, 3,7a-ALX and CSU) showed clear and reproducible effects on the mobility of N-glycosylated ppcecAOPG2 (Figure 4C). Quantification confirmed that all of the compounds had a comparable and statistically significant effect (Figure 4D) and we conclude that all active compounds act at a stage after N-glycan attachment, most likely by targeting α-glycosidase enzymes.

Since the model substrate ppcecAOPG2 may employ both co- and/or post-translational mechanisms of ER translocation, and their associated oligosaccharyltransferase (OST, EC 2.4.99.18) complexes for N-glycosylation (Figures 1A-1B), we compared the results we obtained when the protein was synthesised in the presence (co/post) and absence (post only) of ER-derived microsomes. The quantitative effects of AUS, 3,7,7a-CSU and 3,7-CSU appeared to be more pronounced following post-translational import as compared to the co-translational system (see Figure 4D). However, we note that there is a higher proportion of untranslocated precursor (with the signal sequence intact, see product labelled 0Gly (nc)) following post-translational translocation when compared to the co-translational system (c.f. Figure 4C, lane 2 and Supplementary Figure S1Biv, lane 2, 0Gly (nc) versus 0Gly (sc)). Thus, it may be that there is a reduced level of glycoprotein substrate in the ER lumen following post-translational import thereby allowing even the relatively ineffective compounds AUS, 3,7,7a-CSU and 3,7-CSU to show some degree of inhibitory effect when present at a high concentration (5 mM, see Figure 4D).

The overall conclusion from this quantitative analysis (Figure 4D) was consistent with our previous studies and indicated that CST, DAB, DMDP, 3,7a-ALX and CSU were the most active compounds as judged by changes in glycoprotein mobility. Furthermore, quantification showed that the effects of CST (Figure 2A), KIF (Figure 2B), DAB (Figure 2C), DMDP (Figure 2D), CSU (Figure 2E) and 3,7a-ALX (Figure 2F) were not statistically different between co- and post-translational pathways (Figure 4D). We therefore conclude that the inhibition of N-linked glycan processing

we observe is unaffected by the pathway through which N-glycans are added to the polypeptide chain (cf. Figures 1A-1B).

Active compounds have distinct effects on the activity of purified α-glucosidase II

Thus far, all our data suggest that the effects of the five active compounds observed during the cell-free translation of glycoproteins (CST, DAB, DMDP, 3,7a-ALX and CSU) are due to inhibition of glucose trimming via α-Glu I and/or α-Glu II, both of which are active in the ER lumen (cf. Figure 1). In order to better understand the molecular basis for the effects observed using ER microsomes, we sought to study the activity of the same compounds on the isolated catalytic alpha-subunit of the α-Glu II complex (GIIα) (Figure 5A).

Taking advantage of recent structural studies (Satoh et al. 2016), we expressed and purified the GIIα subunit from the thermophilic fungus *Chaetomium thermophilum* (Figures 5A-5B, Supplementary Table SI) for use in a simple enzyme assay whereby the regulatory β subunit (GIIβ), and its role in N-glycan recognition (Olson et al. 2013), is not required for *in vitro* catalytic activity (Chapdelaine et al. 1978, Trombetta et al. 2001). Using *para*-nitrophenyl-α-*D*-glucopyranoside (PNPG) as a colorimetric substrate (Figure 5C), we estimated values for V_{MAX} of 67.4 ± 0.8 μM/s and K_M of 181 ± 7 μM for *C. thermophilum* GIIα (Figure 5D).

Inclusion of each of our panel of iminosugars (Figure 2) during the enzyme-catalysed reaction showed that CST, DAB and CSU (Figures 2A, 2C and 2E) all strongly inhibited GIIα activity (Figure 5E). In contrast, DMDP (Figure 2D) was a moderate inhibitor and 3,7,7a-CSU (Figure 2H) a very weak inhibitor (Figure 5E). The

compounds KIF, 3,7a-ALX, AUS and 3,7-CSU (Figures 2B, 2F, 2G and 2I) all had no significant effect on enzyme activity (Figure 5E). Of particular interest, is the inactivity of 3,7a-ALX (Figure 2F) towards GIIα since, in the cell-free system, the same compound was effective at inhibiting the ER processing of N-glycans (Figure 4D). As glucose trimming in ER microsomes involves both α-Glu I and α-Glu II, these data suggest that 3,7a-ALX may inhibit glucose trimming via its actions on α-Glu I rather than α-Glu II. This model is also consistent with the glycoprotein profiles of the Op91-2Gly products obtained using 3,7a-ALX and CST (see Figure 3D).

The mode of inhibition of CST, DAB, DMDP and CSU towards GIIα activity was determined using Lineweaver-Burk plots and all four compounds exemplified competitive inhibition (Supplementary Figure S3). Subsequently, K_i values for each compound were calculated from substrate-velocity curves using the Michaelis-Menten model for competitive inhibition (Supplementary Figure S4). Based on these calculations (Table I), we found that DAB is a very effective inhibitor of *C. thermophilum* GIIα (10 fold better than CST), CSU is comparable to CST, whilst DMDP is the weakest inhibitor.

Inhibitors display different interactions with the GIIα active site *in silico*

In order to better understand the differences in the inhibitory activities of DAB, CST, DMDP and CSU, we next modelled these compounds into the substrate binding site of GIIα. To date, two high resolution structures of GIIα have been resolved, one utilising *C. thermophilum* GIIα (Satoh et al. 2016) and the other a murine protein (Caputo et al. 2016). These proteins share 41% and 92% sequence identity respectively with the canine GIIα present in the ER microsomes used in our gel shift

assays (cf. Figures 3-4), and both studies locate the putative enzyme active site in the centre of a highly conserved $(\beta/\alpha)_8$ barrel domain (Supplementary Figure S5). The murine and *C. thermophilum* GIIα proteins have a similar domain architecture and the respective positioning of bound disaccharides is well-matched (Caputo et al. 2018). Given its close similarity to both the canine (92% sequence identity) and human (90% sequence identity) proteins, we utilised the mouse GIIα structure in our docking studies in the hope of obtaining information of potential therapeutic value.

Our modelling studies led us to two clear conclusions; firstly, whilst all four of the compounds that we subjected to a detailed kinetic analysis may form an ionic bond with D564, only the most effective competitive inhibitor, DAB (see Table I), can potentially form a second ionic bond via D640 of the GIIα active site (see Figures 6A-6D and Supplementary Figure S6); secondly, CST and CSU may both form a hydrogen bond with GIIα via H698, which is not formed by DMDP, the least effective inhibitor tested (Table I and Figures 6C-6D versus Figure 6B). Residues D564, D640 and H698 are all conserved between the *C. thermophilum*, canine, murine and human ER α-Glu II enzymes (Supplementary Figure S5, see asterisks and filled circle). We, thus, conclude that the greater inhibitory potency of DAB is driven by an additional ionic interaction formed between the endocyclic nitrogen and GIIα whereas, for the other three compounds, their effectiveness as inhibitors most likely results from small differences in their binding affinity that are driven by their respective hydrogen bonding networks.

Discussion

Here, we have evaluated nine iminosugars as inhibitors of the glycoprotein processing enzymes ER α-Glu I and ER α-Glu II using a combination of two *in vitro* approaches. Firstly, we analysed the effects of compounds on the relative mobility of newly synthesised glycoproteins in the presence of ER microsomes and, secondly, we studied their ability to inhibit purified recombinant GIIα.

ER derived microsomes faithfully recapitulate protein N-glycosylation and subsequent glucose trimming events that occur in the ER lumen but we were unable to detect any evidence of mannose trimming and the commercial cell-free translation system we used precludes any ERAD of the model glycoproteins studied (Vembar and Brodsky 2008, Carlson et al. 2005). Our studies using ER microsomes, thus, allow us to draw two general conclusions in relation to the effects on glucose trimming that we observed with the compounds studied: i) inhibition appears to be independent of the substrate that bears the N-linked glycan(s), the number of glycans present and the location/context of the glycan(s) within the polypeptide; ii) their inhibitory effects are comparable whether N-linked glycans are added co-translationally or post-translationally, most likely via distinct mammalian OST complexes (Ruiz-Canada et al. 2009). On this basis, we conclude that five compounds (CST, DAB, DMDP, CSU and 3,7a-ALX, see Figure 2) inhibit overall glucose trimming in ER derived microsomes. Interestingly, although studies using purified microsomal enzyme fractions suggested AUS is a selective ER α-Glu II inhibitor (Tropea et al. 1989), it resulted in barely detectable levels of inhibition in our cell-free system (Figures 3C, 4C-4D and Supplementary Figure S1). Hence, such gel shift assays may provide a useful *in vitro* tool in the search for broad spectrum

antivirals by helping to identify new compounds that target host ER α-glucosidases (Chang et al. 2013, Alonzi et al. 2017).

Complementary to our studies using ER microsomes, and in an attempt to distinguish between inhibitors of ER α-Glu I/II, we evaluated the same panel of iminosugars (Figure 2) for their ability to inhibit purified α-Glu II. To this end, we expressed and purified the catalytic GIIα subunit from *C. thermophilum* and used it in a simple enzyme assay performed in the presence and absence of the iminosugars. Our preliminary screen of compound activity revealed one very striking result, namely that 3,7a-ALX (Figure 2F) was completely inactive towards GIIα enzyme activity (Figure 5E), despite its effectiveness with ER microsomes (Figure 4D). The simplest explanation for these findings is that 3,7a-ALX is a selective inhibitor of ER α-Glu I and all our data are consistent with this hypothesis. Furthermore, the key residues implicated in substrate binding (Satoh et al. 2016, Caputo et al. 2016, Caputo et al. 2018) are conserved between the *C. thermophilum* and canine alpha-subunits of ER α-Glu II (Supplementary Figure S5), suggesting that our data with the purified enzyme is directly relevant to our microsome based studies. Nevertheless, although we tentatively suggest that 3,7a-ALX may be a selective inhibitor of ER α-Glu I, further experiments, such as studies using purified ER α-Glu I, will be required to confirm this hypothesis.

The four compounds that did inhibit *C. thermophilum* GIIα in our preliminary screen (CST, DAB, DMDP, CSU, see Figure 5E) were subjected to a full kinetic analysis (Supplementary Figures S3-S4), confirming that they all act as competitive inhibitors and allowing us to rank order their effectiveness on the basis of their K_i values (Table

I). In this purified enzyme assay, DAB was over 10-fold more effective than any of the three other compounds tested and we carried out *in silico* modelling of its interaction with the GIIα substrate binding site in order to better understand the structural basis for this enhanced inhibitory activity (Figure 6). Our modelling showed that only DAB has the potential to form a second ionic bond with residue D640 (the catalytic acid/base) of the GIIα active site (Figure 6 and Supplementary Figure S6). We propose that it is this additional ionic bond which enhances the inhibitory activity of DAB over the other three compounds analysed (Supplementary Figure S6). Of the three other inhibitors, we find that CST and CSU are 4 to 7-fold more effective than DMDP, the least effective "active" compound that we tested (Table 1). In this case, our modelling suggests that this variation is based on differences in the number and/or strength of the hydrogen bonding interactions that these three compounds may form with GIIα. Hence, the effectiveness with which DMDP forms hydrogen bonds with the ER α-Glu II substrate binding site is most likely marginally lower than that for CST or CSU (cf. Figure 6).

Despite the differences in their potential bonding interactions within the GIIα active site, all four compounds exhibit K_i values in the mid to low µM range (Table I). Given that inhibition of ER α-Glu I is sufficient but not obligate for antiviral activity (Kiappes et al. 2018), our kinetic and *in silico* characterisation of CST, DAB, DMDP and CSU as inhibitors of ER α-Glu II presents a potential platform for the development of therapeutic antivirals targeting host ER α-glucosidases. Interestingly, the most selective ER α-Glu II inhibitor to date, the recently identified DNJ-tocopherol conjugate (ToP-DNJ) (Kiappes et al. 2018), is a second-generation derivative of DNJ. Hence, incorporation of the tocopherol moiety into the iminosugar scaffold of CST,

DAB, DMDP or CSU may yield an iminosugar-conjugate with increased selectivity towards ER α-Glu II (cf. Kiappes et al. 2018).

Furthermore, whilst antiviral activity resulting from inhibition of ER α-Glu I/II has been attributed to abrogation of glucose trimming and failure to enter and/or exit the CNX-CRT cycle, it is also conceivable that, through inhibition of ER α-Glu II, glycoproteins may participate in an alternative, and mechanistically distinct, protein quality control pathway involving the carbohydrate binding protein malectin (Figure 1C), which specifically associates with di-glucosylated N-glycans (Schallus et al. 2008, Galli et al. 2011). Malectin preferentially binds misfolded ERAD substrates as opposed to partially/correctly folded glycoproteins (Chen et al. 2011). However, although the malectin-glycoprotein association is G2M9-dependent, a G2M9 N-glycan alone is not sufficient to selectively distinguish between glycoproteins based on their level of folding (Qin et al. 2012). Instead, it is proposed that a complex formed by malectin and the ribophorin I subunit of the OST (cf. Figure 1) exerts glycoprotein quality control for these substrates (Quin et al. 2012, Yang et al. 2018). Thus, by selectively inhibiting ER α-Glu II the resulting accumulation of di-glucosylated N-glycan species may lead to an increased association with the malectin-ribophorin I complex as well as a stalling of the CNX-CRT cycle, thereby disrupting viral glycoprotein folding through two mechanistically distinct quality control pathways.

In summary, we have utilised a cell-free system which recapitulates N-glycosylation events in the ER, performed kinetic studies with purified GIIα and employed a docking model of the GIIα active site to characterise a panel iminosugars as inhibitors of the glycoprotein processing enzymes ER α-Glu I and ER α-Glu II. Our

study extends the chemical space surrounding ER α-Glu I/II iminosugar inhibitors whereby we identify the C-3 substituted pyrrolizidines CSU and 3,7a-ALX as promising second-generation iminosugars.
