# Supplementary material for: Characterizing the selectivity of ER α-glucosidase inhibitors
Source: Glycobiology. 2019 Apr 29;29(7):530–42. doi: 10.1093/glycob/cwz029 (PMC6583763; doi:10.1093/glycob/cwz029)
Supplement: Supplementary Data [file cwz029_supplementary_data_13.3.19.pdf]

Characterising the selectivity of ER α-glucosidase inhibitors

Supplementary Data: Figures S1-S6 and Table SI

Figure S1 *The effects of active compounds are independent of glycoprotein substrate*

Figure S2 *Migration profiles of glycoprotein species generated by active compounds*

Table SI *Mass spectrometric analysis of GIIα (C. thermophilum)*

Figure S3 *Active compounds are competitive inhibitors of ER α-glucosidase II*

Figure S4 *K_i calculations for ER α-glucosidase II inhibitors*

Figure S5 *Alignment of ER α-glucosidase II α-subunit sequences*

Figure S6 *Bonding between active inhibitors and residue D_{640} of mouse GIIα*

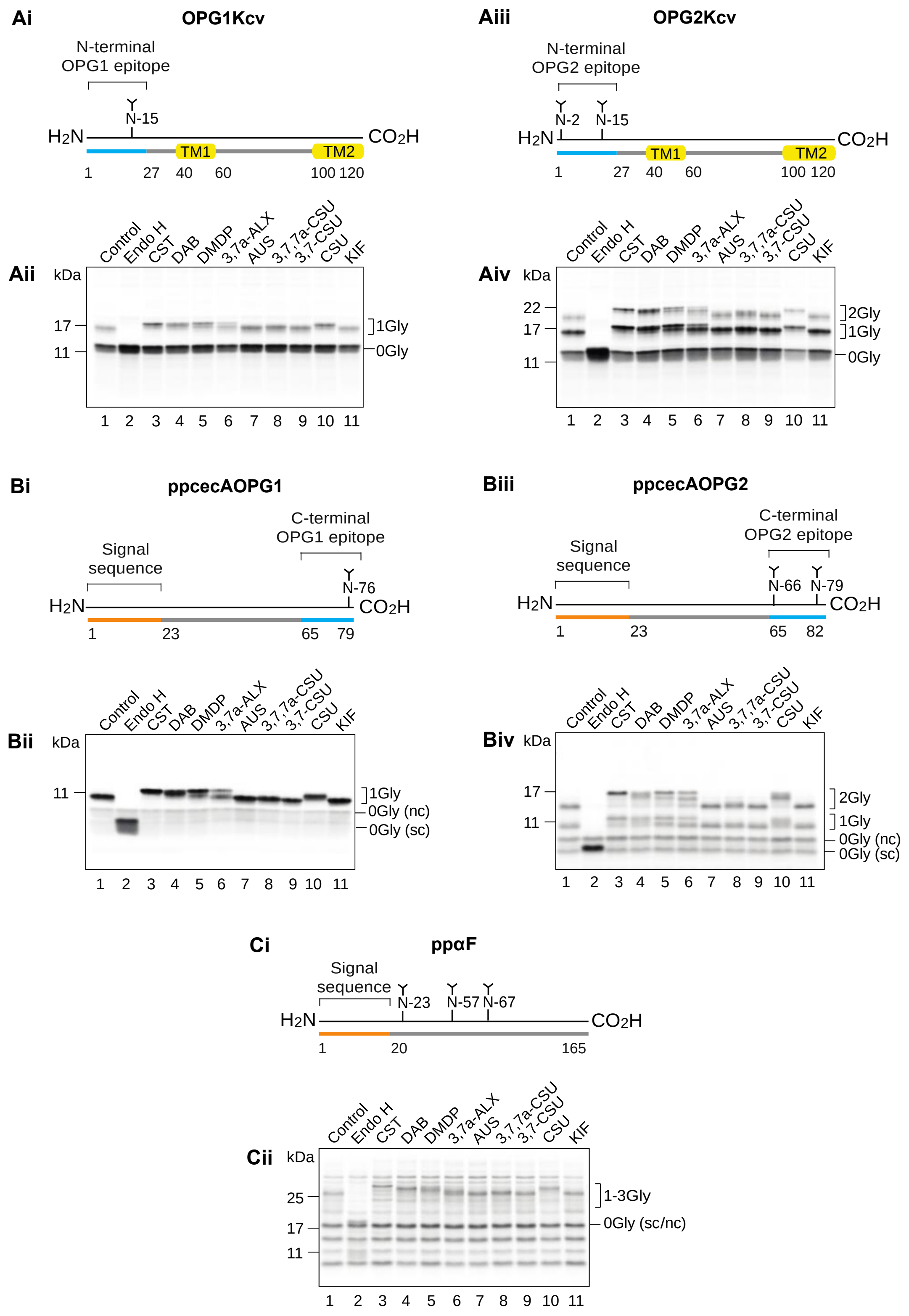

Figure S1*. The effects of active compounds are independent of glycoprotein substrate.* Protein substrates were synthesised in the presence, or absence, of the compounds indicated (5 mM) using the co-translational system as described in the

legend to Figure 3 (see main text) and radiolabelled products analysed by SDS-PAGE. Substrates examined were: (**Ai, Aii**) OPG1Kcv; potassium channel protein Kcv modified with an N-terminal reporter containing one N-glycosylation site (residues 1-26 of bovine rhodopsin, residue T4 mutated to A) and two transmembrane domains (TM1 and TM2), (**Aiii, Aiv**) OPG2Kcv, potassium channel protein Kcv modified with an N-terminal reporter with both N-glycosylation sites intact (Watson et al. 2013), (**Bi, Bii**) ppcecAOPG1, preprocecropin A modified with a C-terminal reporter (a 14 residue N-terminal fragment of bovine rhodopsin) containing one N-glycosylation site (Johnson et al. 2012), (**Biii, Biv**) ppcecAOPG2 (cf. Figure 3B), preprocecropin A modified with a C-terminal reporter (residues 1-18 of bovine rhodopsin) containing two N-glycosylation sites, (**Ci, Cii**) ppαF, the yeast secretory protein prepro-alpha-factor comprised of a signal sequence and three endogenous sites for N-glycosylation (N23, N57 and N67); nc, non-cleaved signal sequence forms of ppcecAOPG1, ppcecAOPG2 or ppαF; sc, signal cleaved forms.

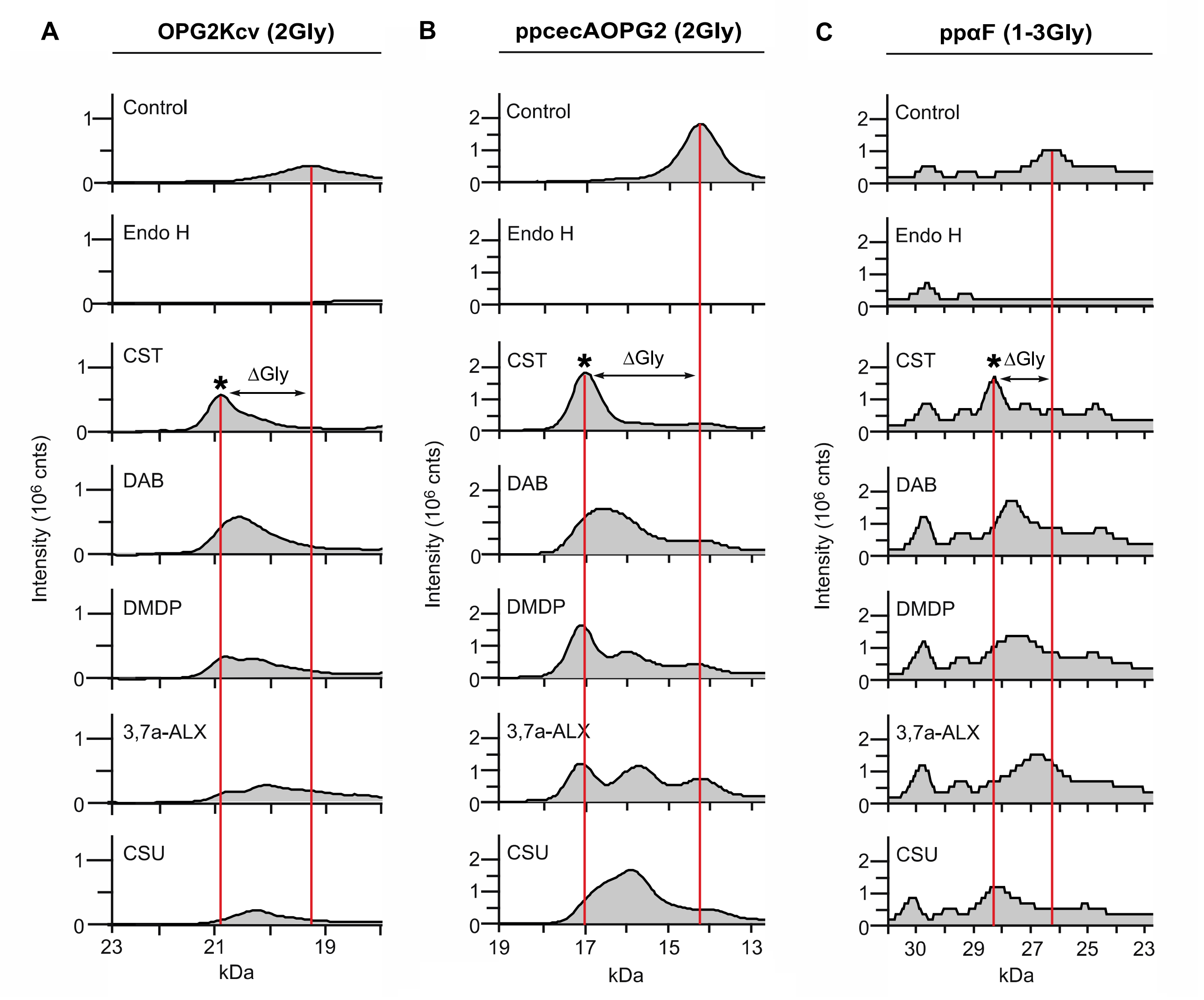

Figure S2. ***Migration profiles of glycoprotein species generated by active compounds.*** The gel shifts of the N-glycosylated species of (**A**) OPG2Kcv, (**B**) ppcecAOPG2 and (**C**) prepro-alpha-factor (ppαF) in Supplementary Figure S1 (see Aiv, Biv, Cii lanes 1, 2, 3, 4, 5, 6 and 10) were analysed as described in the legend to Figure 3 (see main text). Migration profiles were generated using the doubly N-glycosylated species with the exception of ppαF as the 1-3 Gly forms were not distinctly distinguishable from each other. ΔGly depicts alterations in N-glycan trimming as judged by changes in glycoprotein mobility, with the G3M9 N-glycan form (based on CST treatment) denoted by an asterisk (*).

Table SI. ***Mass spectrometric analysis of GIIα (C. thermophilum)***

Identified Proteins	Unique Peptides	Total Spectra	Sequence Coverage (%)	Mw (kDa)
[a] GIIα: OS=*C. thermophilum* GN=CTHT_0064960 PE=1 SV=1	89	1247	78	111
[a] Serum albumin: OS=*Bos taurus* GN=ALB PE=1 SV=4	3	3	6	69
[b] 2-oxoglutarate dehydrogenase decarboxylase component: OS=*E. coli* D6-113.11 GN=sucA PE=4 SV=1	9	10	13	105
[b] Aconitate hydratase B: OS=*E. coli* 1-176-05_S3_C2 GN=acnB PE=3 SV=1	4	4	6	91
[b] Valine--tRNA ligase: OS=*E. coli* GN=valS PE=3 SV=1	5	5	6	108
[b] Aconitate hydratase: OS=*E. coli* 1-176-05_S3_C2 GN=acnA PE=3 SV=1	3	3	3	98

[a] Database: BioMS, University of Manchester, [b] Database: *Swiss*prot and Trembl; Protein Threshold: 95%; Minimum number of Peptides: 2; Peptide Threshold: 50%; Identified proteins are named according to the FASTA format wherein: OS = organism name, GN = gene name, PE = protein existence, SV = sequence version.

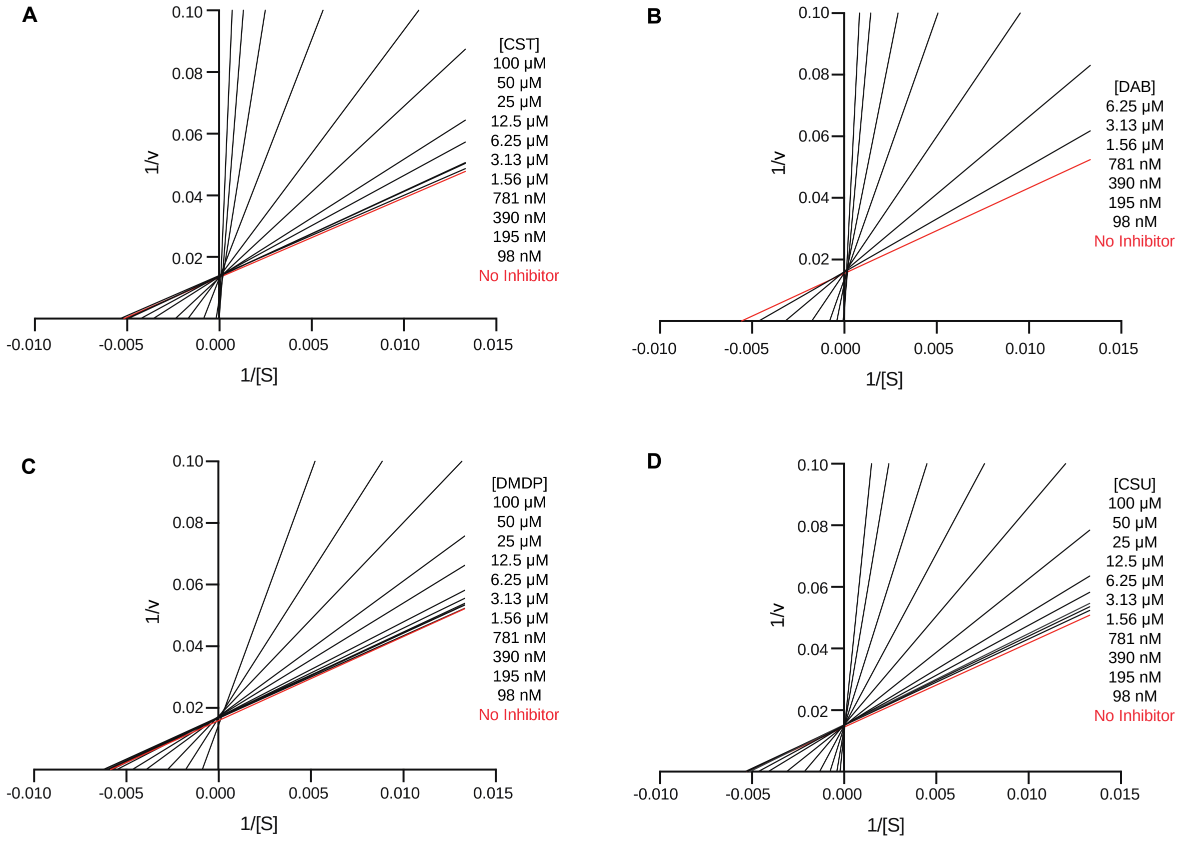

Figure S3. ***Active compounds are competitive inhibitors of ER α-glucosidase II.*** PNPG (2 mM, 1.5 mM, 1 mM, 500 μM, 350 μM, 200 μM, 150 μM, 125 μM, 100 μM and 75 μM) and inhibitor, at varying concentrations as indicated, were incubated with GIIα (6 μg/mL) at 37°C and absorbance measurements (λ = 410 nm, 1 min intervals, 90 min) used to generate Lineweaver-Burk plots for (**A**) CST, (**B**) DAB, (**C**) DMDP, (**D**), CSU. Enzyme activity assays were performed in triplicate (*n*=3). CST, DAB, DMDP and CSU competitively inhibit ER α-glucosidase II.

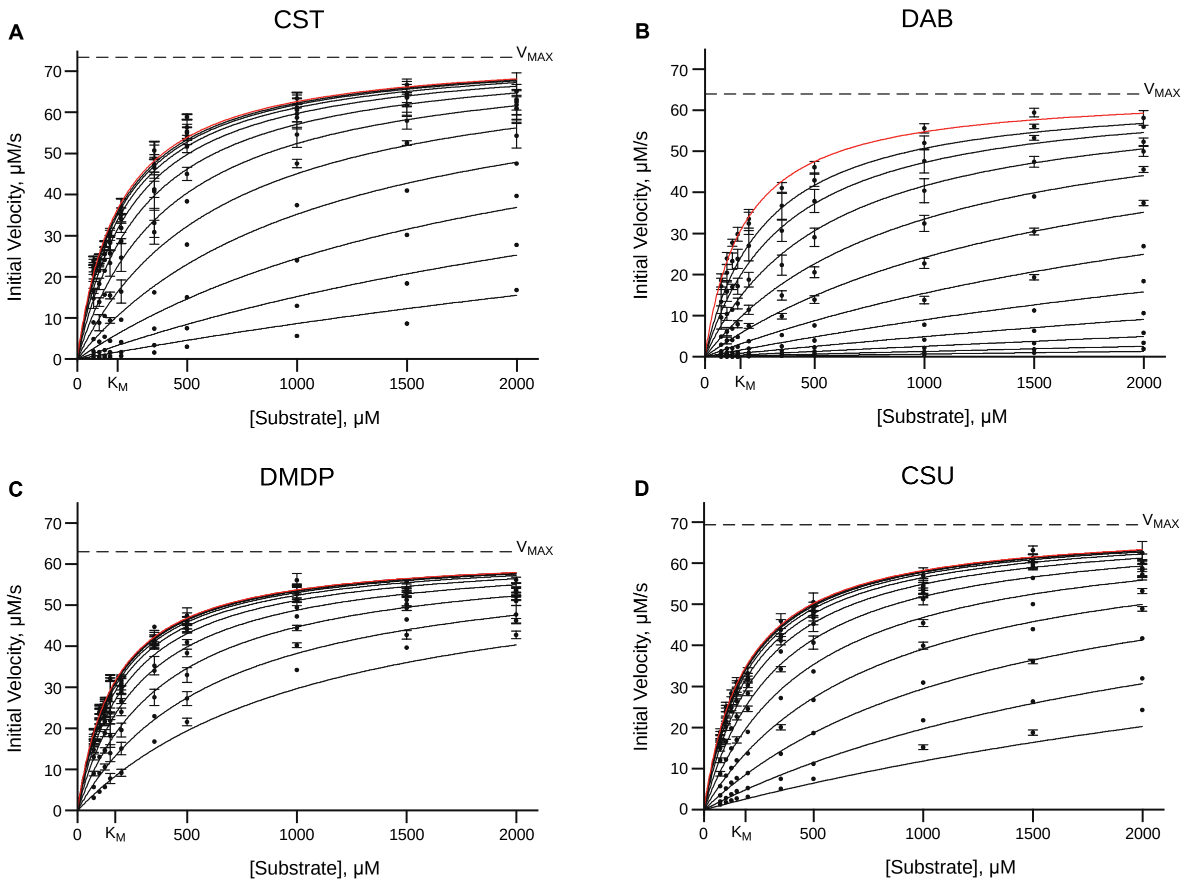

Figure S4. ***K_i* *calculations for ER α-glucosidase II inhibitors.***

The data from the experiments described in the legend to Supplementary Figure S3, together with the values for concentrations of DAB greater than 6.25 μM that were not included in Supplementary Figure S3B, were used to generate substrate-velocity curves (*n*=3) and K_i values estimated using the Michaelis-Menten model for competitive inhibition. The resulting values were (**A**) CST; K_i 2.60 ± 0.58 μM, V_{MAX} 74.72 ± 1.83 μM/s, K_M 192.8 ± 14.12 μM, R^2 = 0.982, (**B**) DAB; K_i 0.187 ± 0.003 μM, V_{MAX} 64.61 ± 0.82 μM/s, K_M 179.4 ± 7.43 μM, R^2 = 0.991, (**C**) DMDP; K_i 18.07 ± 0.51 μM, V_{MAX} 62.98 ± 1.1 μM/s, K_M 171.4 ± 10.12 μM, R^2 = 0.973, (**D**) CSU; K_i 4.07 ± 0.06 μM, V_{MAX} 69.35 ± 0.89 μM/s, K_M 189.3 ± 7.77 μM, R^2 = 0.991. Data was fitted using V_{MAX} and K_M values obtained from non-inhibitor controls measured at the same time as inhibitor samples for each compound.

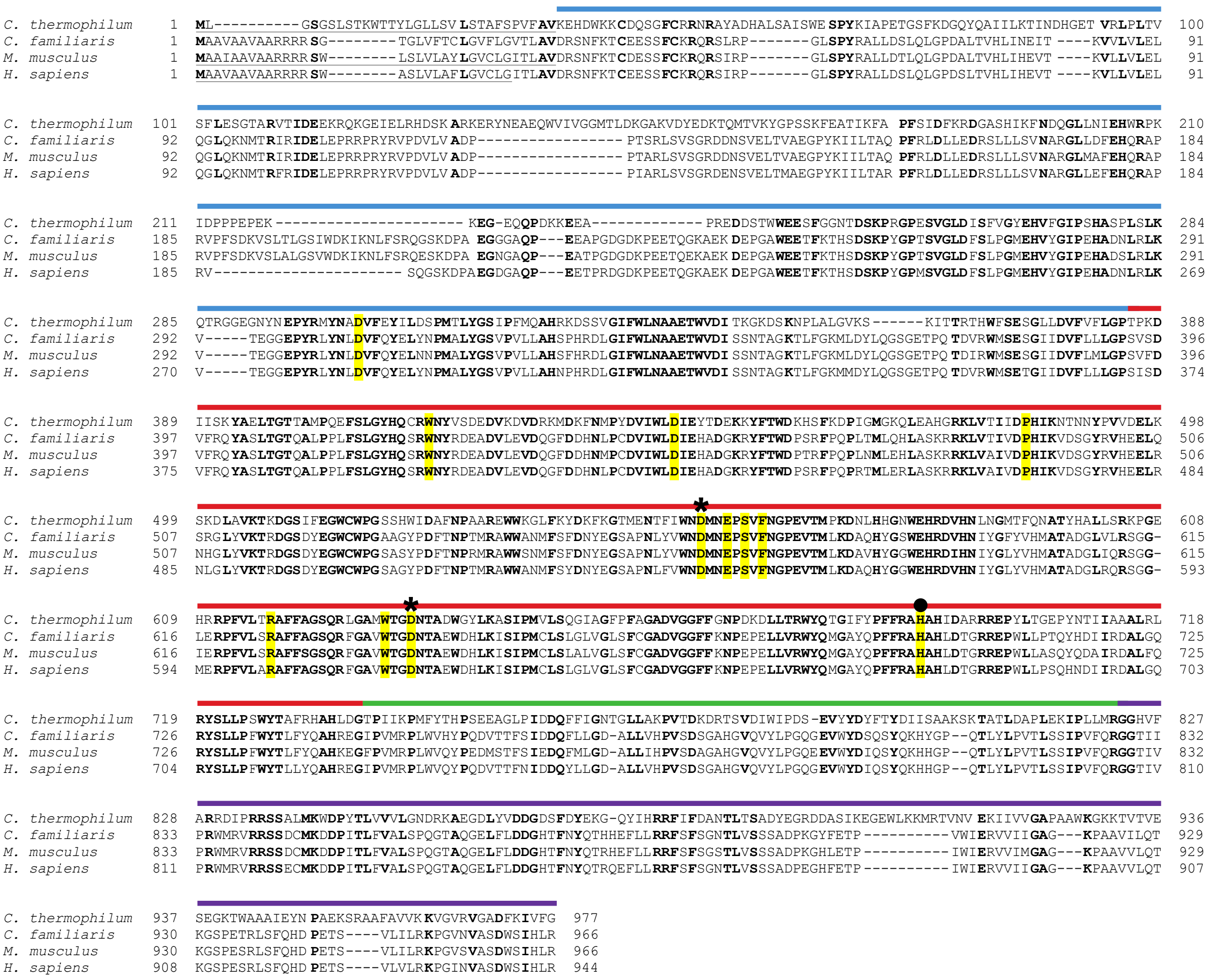

Figure S5. ***Alignment of ER α-glucosidase II α-subunit sequences.***

The amino acid sequences of the ER GIIα subunit of fungal (*C. thermophilum*, G0SG42), canine (*C. familiaris,* E2R729), murine (*M. musculus*, Isoform 2, Q8BHN3) and human (*H. sapiens*, Isoform 1, Q14697) origin were aligned using PROMALS3D. N-terminal signal sequences that are cleaved from the mature protein are underlined (*C. familiaris* signal sequence not included on Uniprot), whilst other subdomains are colour coded as for Figure 5A (see main text); i.e. N-terminal domain (blue), $(\beta/\alpha)_8$ domain (red), proximal C-terminal domain (green), distal C-terminal domain (purple). Residues conserved between all four species are in bold, whilst those implicated in substrate binding are highlighted in yellow. Residues D564 and D640 which are

implicated in forming in ionic interactions are indicated by asterisks (*) whilst residue H698 implicated in hydrogen bonding is indicated by a filled circle (•). These residues are given according to numbering of the murine sequence and are discussed in the main text.

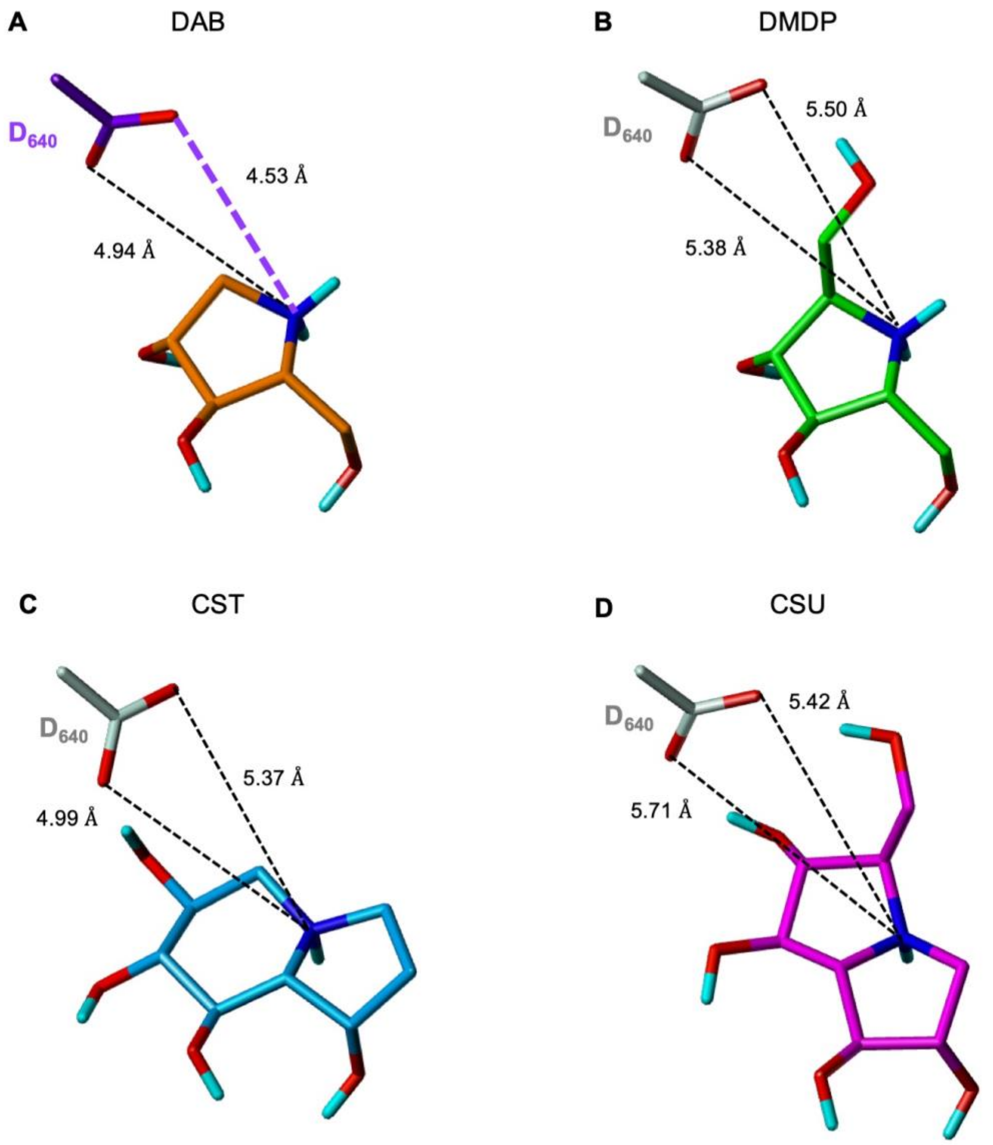

Figure S6. ***Bonding between active inhibitors and residue D_{640} of mouse GIIα.*** The estimated distance between the endocyclic nitrogen of the four active inhibitors and residue D640 of murine GIIα was calculated by *in silico* modelling (see materials and methods). In the case of DAB (**A**) the calculated distance is short enough to potentially form an ionic interaction (purple dashed line). For the other three compounds studied, DMDP (**B**), CST (**C**) and CSU (**D**), their endocyclic nitrogen atoms are located further away from residue D640 thereby precluding any ionic interaction (black dashed lines).
